# Synthesis and Pharmacological Characterization of Visabron, a Backbone Cyclic Peptide Dual Antagonist of α4β1 (VLA-4)/α9β1 Integrin for Therapy of Multiple Sclerosis

**DOI:** 10.1021/jacsau.1c00496

**Published:** 2021-11-24

**Authors:** Chaim Gilon, Michal Klazas, Adi Lahiani, Adi Schumacher-Klinger, Shira Merzbach, Johnny N. Naoum, Haim Ovadia, Limor Rubin, Susan Cornell-Kennon, Erik M. Schaefer, Jehoshua Katzhendler, Cezary Marcinkiewicz, Amnon Hoffman, Philip Lazarovici

**Affiliations:** $ Institute of Chemistry, The Hebrew University of Jerusalem, Jerusalem 91904, Israel; ^@^Pharmacy, ^‡^Pharmacology, and ^∥^Medicinal Chemistry, Institute for Drug Research, School of Pharmacy, Faculty of Medicine, The Hebrew University of Jerusalem, Jerusalem 9112102, Israel; ^§^Neurology and ^&^Allergy and Clinical Immunology Unit, Hadassah-Hebrew University Medical Center, Jerusalem 9112001, Israel; + AssayQuant Technologies, Inc., 260 Cedar Hill Street, Marlboro, Massachusetts 01752, United States; ¶ Department of Bioengineering, College of Engineering, Temple University, Philadelphia, Pennsylvania 19122, United States

**Keywords:** α4β1, α9β1, backbone cyclic TMLD peptide, disintegrin, EAE mice model, integrin-overexpressor cells, immunogenicity, lymphocytes, multiple sclerosis, macrophage, natalizumab, off-target, pharmacokinetics, safety, selectivity, serum stability, solid-phase peptide synthesis

## Abstract

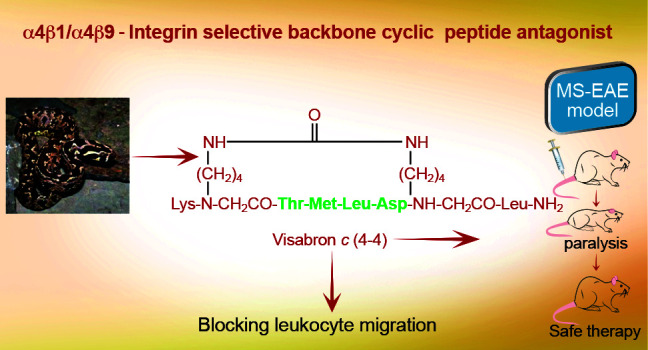

Integrins α4β1/ α9β1 are important in the pathogenesis and progression of inflammatory and autoimmune diseases by their roles in leukocyte activation and trafficking. Natalizumab, a monoclonal antibody selectively targeting α4β1 integrin and blocking leukocyte trafficking to the central nervous system, is an immunotherapy for multiple sclerosis (MS). However, due to its adverse effects associated with chronic treatment, alternative strategies using small peptide mimetic inhibitors are being sought. In the present study, we synthesized and characterized visabron *c* (4–4), a backbone cyclic octapeptide based on the sequence TMLD, a non-RGD unique α4β1 integrin recognition sequence motif derived from visabres, a proteinous disintegrin from the viper venom. Visabron *c* (4–4) was selected from a minilibrary with conformational diversity based on its potency and selectivity in functional adhesion cellular assays. Visabron *c* (4–4)’s serum stability, pharmacokinetics, and therapeutic effects following ip injection were assessed in an experimental autoimmune encephalomyelitis (EAE) animal model. Furthermore, visabron *c* (4–4)’s lack of toxic effects in mice was verified by blood analysis, tissue pathology, immunogenicity, and “off-target” effects, indicating its significant tolerability and lack of immunogenicity. Visabron *c* (4–4) can be delivered systemically. The *in vitro* and *in vivo* data justify visabron *c* (4–4) as a safe alternative peptidomimetic lead compound/drug to monoclonal anti-α4 integrin antibodies, steroids, and other immunosuppressant drugs. Moreover, visabron *c* (4–4) design may pave the way for developing new therapies for a variety of other inflammatory and/or autoimmune diseases.

## Introduction

Integrins are a family of cell surface receptors composed of noncovalently linked α and β subunits that regulate many biological functions in eukaryotic cells and play essential roles in regulating cell adhesion and migration.^1^ Each of the α–β heterodimer integrins exhibits a distinct ligand-binding profile and pairs with one of the β1−β7 subunits. The α4β1 and α4β7 integrins have been established as the most selective receptors for fibronectin, vascular adhesion molecule (VCAM-1), and mucosal addressin cell adhesion molecule-1 (MdCAM-1) and have been recognized as important targets for the development of monoclonal antibodies, peptidomimetics, and small molecules for therapy of multiple sclerosis and inflammatory diseases of the digestive system.^2,3^ The α4β1 (also known as very late antigen-4 (VLA-4) or CD49d/CD29), α4β7, and α9β1 integrins bind to an acidic motif termed “LDV” in fibronectin. They share several structural and functional properties, have similar tissue expression profiles, and are classified into a common subgroup within the integrin family. The cytoplasmic domains of the α4 and α9 integrins share about 40% amino acid sequence identity.^4^ These integrins are important regulators of cytoskeletal dynamics in cell adhesion and migration. They are overexpressed in pathological states such as wounds, inflammation, autoimmune diseases, and cancer, making these receptors important therapeutic targets for many inflammatory diseases.^5^

Multiple sclerosis (MS) is a central nervous system (CNS) neurological disorder generally viewed as having an autoimmune origin. Likewise, in other various inflammatory diseases, including asthma, rheumatoid arthritis, and inflammatory bowel disease (IBD), the binding of the leukocytes α4β1 integrin to VCAM-1 ligand expressed on endothelial cells initiates adhesion of the leukocyte to the vascular endothelium followed by extravasation into the CNS tissue, thus contributing to the inflammation and pathogenesis of the MS disease. Treatment with the monoclonal antibodies natalizumab (Tysabri) for therapy of MS and vedolizumab (Entyvio) for IBD, which inhibit α4β1 and α4β7, respectively, has been found to be efficient in the clinic for therapy of chronic relapsing inflammation of demyelinating MS disorders and treatment of resistant Crohn’s disease.^6^ However, the therapeutic efficacy of natalizumab is associated with adverse effects.^7^ Moreover, neutralizing antibodies toward natalizumab that cause loss of drug efficacy have been identified in about 6% of patients. The greatest risk of natalizumab treatment is associated with the development of progressive multifocal leukoencephalopathy (PML), a rare but serious viral infection (JC-virus) leading to inflammation and demyelination, resulting in severe disability or death.^8^ Because of the risks associated with natalizumab and its biosimilar antibodies, there are many reservations over their use in the clinic as a preferred treatment option. Although antibodies and small molecules have been developed to inhibit α4β1/α4β7 integrins with potency and selectivity, there is an unmet clinical need for the development of cheaper, effective α4β1/α4β7 peptidomimetic antagonists with improved safety and pharmacokinetics for the therapy of inflammatory and autoimmune diseases. Moreover, most of the known compounds are either dual antagonists of α4β1/α4β7 integrins or are selective for α4β7, while very few compounds are selective for α4β1. Peptide lead compounds containing the amino acid motif LDTSL (Leu-Asp-Thr-Ser-Leu) or their truncated versions such as Asp-Thr and Leu-Asp-Thr were found to bind α4β7 and inhibit adhesion of leukocytes, expressing α4β7 to the MAdCAM-1 ligand.^3^ A library of conformationally restricted end-to-end cyclic hexapeptides was synthesized, presenting the pharmacophore Leu-Asp-Thr (LDT) sequence in different conformations, indicating potent and selective α4β7 integrin antagonism of MAdCAM-1 binding to the α4β7 integrin.^9^ Mannose-based peptidomimetics selectively blocking MAdCAM-1 were also developed.^10^ In another approach, PTG-100 and its PN-943 analogue, an oral α4β7 integrin peptide antagonist, were developed to treat inflammatory bowel disease. In addition, high-affinity, selective BIO-1211 inhibitors of α4β1, based on the Leu-Asp-Val (LDV) sequence^11^ and small, end-to-end cyclic Ile-Leu-Asp-Val (ILDV) peptides, were also described.^12^

The present approach for the development of antagonists for α4β1/α4β7 integrins is based on the identification of novel amino acid sequence motifs targeting α4β1/α4β7 integrins present in snake venom disintegrins. These are a family of low-molecular-weight, cysteine-rich proteins that potently and selectively target and inhibit the α4β1/α4β7 integrins.^13,14^ Thereafter, our strategy was to transform the linear peptide motif into an active backbone-cyclic peptide with improved drug properties. Backbone cyclization (BC) was already proven a valuable tool in the methodological conversion of active regions of proteins to cyclic peptidomimetic drug leads.^15,16^ The BC method is employed to introduce global constraints to peptides and active regions in proteins. It differs from other cyclization methods since it utilizes the amide bond nitrogen to anchor the cyclizing bridge, thus maintaining the active pharmacophore. It is based on the incorporation of *N*-alkylated, epsilon-functionalized amino acids into the active sequence for cyclization. BC proved superior to other stabilization methods since the resultant peptides had defined structures that led to better selectivity^17^ and improved pharmacological properties.^15^ However, obtaining the desired active cyclic analogue based on a linear sequence is not a straightforward process and, therefore, requires selection from designed libraries with conformational diversity.^15^ In this study, we describe the isolation of visabres disintegrin from viper venom, identification of the TMLD motif selective for α4β1/α4β7 integrin inhibition, and attempts for its cyclization to generate visabron, a backbone cyclic peptide dual antagonist of α4β1 (VLA-4)/ α9β1 integrin with druglike properties. The pharmacological profile of visabron was achieved by *in vitro* functional cell adhesion and selectivity assays, characterizations of its pharmacokinetic, and safety properties and measurement of its therapeutic effect *in vivo* in a multiple sclerosis–experimental autoimmune encephalomyelitis mouse model.

## Results and Discussion

### Purification and Structure Evaluation of Visabres, an α4-Antagonist from *Vipera daboia* Venom

The first step of the isolation of visabres was based on the preparative fractionation of the venom by FPLC gel filtration^18^ into three protein fractions (Figure S1-A). Fraction Vd-III, which was less toxic, not hemorrhagic, and less contaminated with protease and phospholipase A_2_ activity but enriched in α4-inhibitory activity (Tables S1 and S2), was further separated by a consecutive step of reversed-phase HPLC using a linear gradient of increasing acetonitrile concentration (Figure S1-B). The α4-inhibitory disintegrin fraction, named visabres, was identified by inhibition of Jurkat cell adhesion to human recombinant VCAM-1 (Figure S1-A). Mass spectroscopy analyses of this fraction indicated it is enriched in disintegrins but still contaminated with other venom proteins (Table S3). Final purification of visabres was achieved by a consecutive, similar HPLC separation resulting in a pure α4-disintegrin eluted as a single peak at an acetonitrile concentration of ∼43% (Figure S1-C). The purity of visabres was confirmed by SDS-PAGE in reduced and nonreduced conditions, indicating the dimeric structure of this molecule (Figure S1-D). The amino acid sequence of visabres (Table S4) was determined using a standard procedure, previously applied for other dimeric snake venom disintegrins.^19^ Alignment of the sequence of the α4/α9-integrin-binding motif in visabres subunit B, compared to other dimeric disintegrins (Table 1), indicated a very high homology in the integrin-binding loop C**KRTMLDGLNDY**C that contains the **T**^**41**^**MLD**^**44**^ core motif, obligatory for binding and selectivity toward α4-integrin family.^13^ Visabres, like the TMLD-disintegrin VLO5, was found to be highly potent in nanomolar concentrations and very selective, a dual antagonist of α4β1/α9β1 integrin (Table S5, Figure S2). Visabres attenuated by about 50% the clinical neurological score in EAE mice, upon i.p. injection, using a nontoxic, cumulative dose of 2.5 mg/kg (data not shown).

**Table 1:**
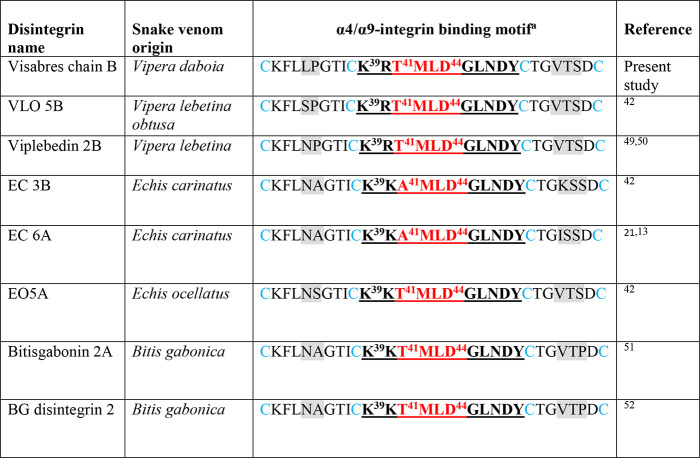
α4/α9-Integrin Binding Motif in Visabres Compared to Other Dimeric Disintegrins

a α4/α9-integrin binding motif is underlined; gray shadowed areas represent variable amino acids among the disintegrins protein family.

### Development of Visabron, an α4-Antagonist Lead Compound Based on the Visabres TMLD-Pharmacophore Motif

**Figure 1:**
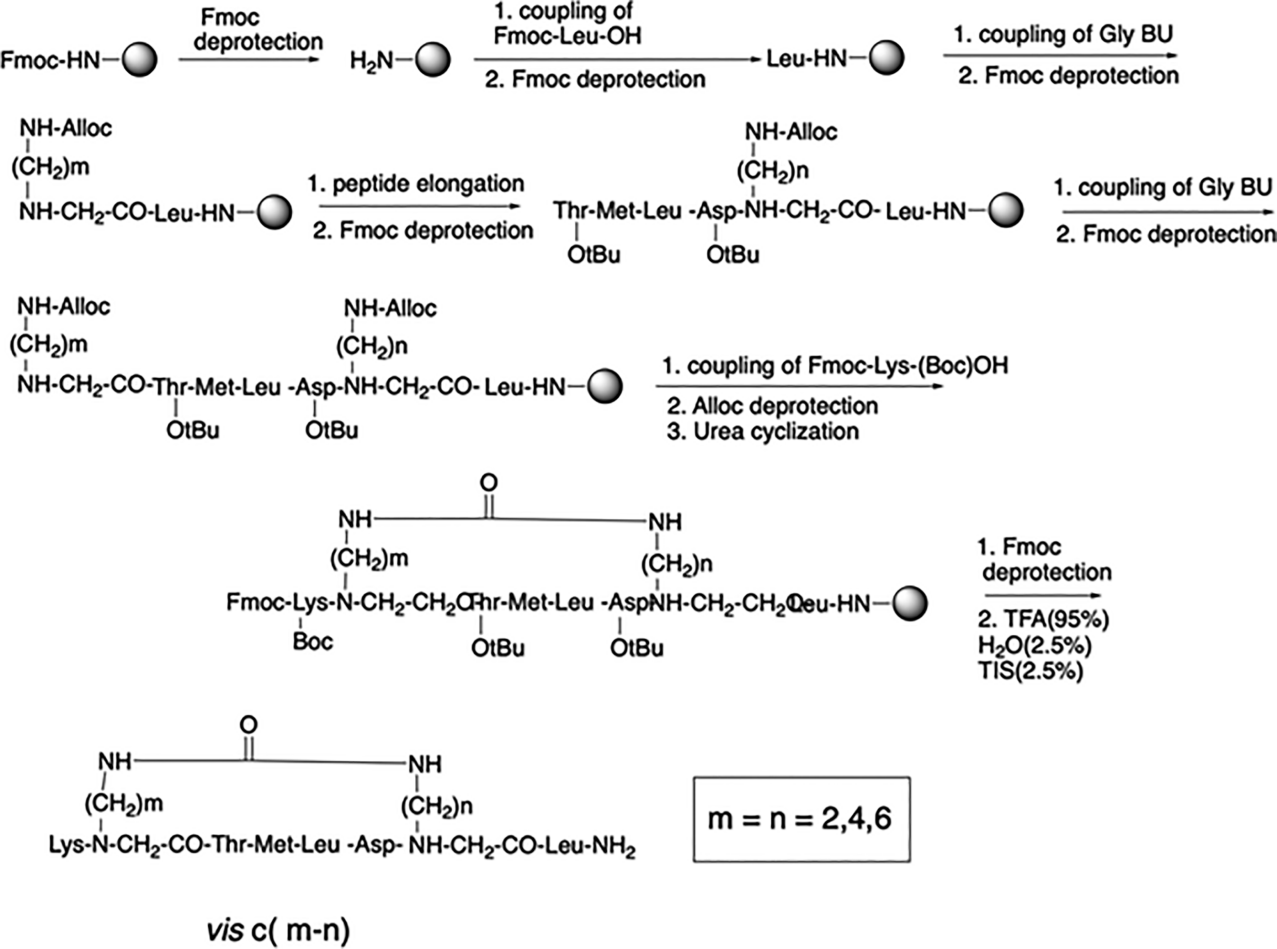
Synthetic scheme for preparing visabrons *c* (*m*–*n*) by solid-phase peptide synthesis.

At first, we began the lead peptide development by solid-phase peptide synthesis (SPPS) and screening for competition in adhesion binding to α4β1/α9β1 compared to α1/α2/α5-β1 integrin of two linear peptides of 31 and 27 amino acids (Table S6, sequences 1 and 2 respectively), covering part of the sequence of chain B of visabres (Table S4). It was found that the TMLD-containing linear peptide was important in the recognition and inhibition of the α4β1 integrin (Table S6, sequence 1). Thereafter, by removing amino acids from the amino and carboxy terminals of this linear peptide, in order to generate a small peptide with druglike properties with molecular weight around 1000 Da,^20^ we found that the linear pharmacophoric KRTMLDGL sequence is selective for inhibition of α4β1/α9β1 integrin,^21^ albeit of very low potency (Table S6, sequence 5). Additional work indicated that replacing threonine in position 41 to alanine, as found in some natural disintegrins (Table 1, EC 3B and 6A), reduced activity (Table S6, sequence 4), further indicating that the TMLD “hot spot” in the integrin-binding loop, at the exact position as the RGD motif present in other disintegrins,^13^ is important for the integrin-binding affinity. In the past, we prepared different cyclic peptides with cysteine bridges at different positions, in which the KRTMLDGL sequence was stabilized and folded. However, although some of these peptides selectively inhibited α4β1 integrin-mediated cell adhesion, they suffered from poor solubility and stability in aqueous solutions (data not shown). Therefore, the present rational for the synthesis of a visabres-derived backbone cyclic peptide named visbron was to replace the visabres arginine at position 40 by glycine (a modification which did not affect the inhibitory activity of the linear peptide KRTMLDGL toward α4β1/α9β1 integrin (Table S6, sequence 6)) by forming a ring between the amide bond nitrogens of the two glycines at positions 40 and 45. Thus, an N-alkylated glycine-building unit (Figure S3) replaced the arginine residue in visabron enabling backbone cyclization (Figure 1). On the basis of these considerations and starting from the general basic synthetic scheme in which the bridge can be of a different size, depending on the number of the methylene groups (*n* and *m* length),^22^ a minilibrary was prepared. Three backbone cyclic analogues were synthesized by SPPS and purified by preparative HPLC with high yields, named visabron *c* (2–2), *c* (4–4), and *c* (6–6), with ring sizes of 23, 27, and 31 atoms, respectively (Figure 1). All members of this library have identical sequences. Analytical HPLC (Figure S4) and MS characterizations (Figure.S5) of the synthesized backbone cyclic visabron peptides indicated 95–99% purity and molecular weights of 945, 1001, and 1057 Da, respectively (Table S7). The visabron peptides were characterized by very good aqueous solubility as also reflected by their cLogP, which is smaller than 5.0 (Table S8), a property important for a drug to be administrated orally or by injection.^23^ Visabron is a polar peptide with a strong positive charge (lysine, K^39^) on one end, a property that is required for the α4β1/α9β1 integrin-binding affinity, considering that substitution of lysine^39^ to arginine in Arg-visabron *c* (4–4) (Figure S6) caused significant loss of activity (Table 2). The ELISA binding experiments clearly indicated the inability of visabrons to antagonize α1, α2, and α5 integrin-mediated adhesion to collagen IV, I, and fibronectin, confirming their high selectivity for α4/ α9 integrins. The binding selectivity to immobilized human recombinant VCAM-1, but not MAdCAM-1, further indicated the ability of visabrons to differentiate between the integrin α4β1 compared to α4β7 (Table 2). Dose–response in cell adhesion assays using Jurkat lymphocytes and U937 macrophages quantitatively estimated the different, dual visabrons antagonistic potency toward α4/α9-β1 integrin-mediated adhesion to immobilized VCAM-1, indicating the following order of potency: visabron *c* (4–4) > visabron *c*(6–6) > visabron *c* (2–2) (Table 2). The potency of visabron was in the low micromolar range in contrast to the poor millimolar potency of linear peptides and the nanomolar very high potency of the parental disintegrin visabres and the drug natalizumab. This indicates that visabron conformation is important in targeting the binding pocket of the integrin. However, improved conformation and/or additional motifs, enabling multivalent interactions with the α4/α9 integrin, may be required to generate very high affinity and potency.^24^ Although cell expression of α4β1 is constitutive, its interaction with ligands is strongly enhanced in an activated state that can be induced by various stimuli, including pro-inflammatory cytokines, phorbol esters, etc.,^25^ affecting the *in vivo* potency of the drug. For this reason, visabrons’ antagonism potency was investigated toward Jurkat lymphocytes and U937 macrophages treated with either TNFα or PMA and found to be enhanced compared to untreated cells (Table 2), a pharmacodynamic property relevant for the ***in vivo*** therapeutic effect. The results of visabron’s antagonism (Table 2) suggested that the conformation of the TMLD motif closely resembles the integrin receptor-bound conformation. The lack of activity of the linear TMLD sequence (Table S6) emphasized that the activity of the visabron cyclic peptides was derived from a conformational effect. Therefore, we chose the most active backbone cyclic peptide visabron *c* (4–4) as the lead structure for further pharmacological characterizations.

**Table 2:** Visabron Analogues’ Potency and Selectivity of Inhibition of Adhesion of Integrin Overexpressor Cells, Lymphocytes, and Macrophages and in ELISA Binding Assay toward the Respective Extracellular Matrix-Immobilized Protein Ligands^a^

							Jurkat lymphocytes-VACAM-1^c^		U937 macrophages-VCAM-1^d^	
peptide IC_50_(μM)	α1β1-collagen IV	α2β1-collagen I	α5β1-fibronectin	α4β1-VCAM-1 F_c_ chimera^b^	α4β7-MAdCAM-1 F_c_ chimera^b^	α9β1-VCAM-1 F_c_ chimera^b^	control	TNFα	control	PMA
natalizumab	>10000	>10000	>10000	0.010	0.015	0.015	0.005	0.002	nt	nt
visabres	>5000	>5000	>3500	0.004	0.200	0.025	0.022	0.015	0.0005	0.0025
visabron *c* (2–2)	>10000	>10000	>10000	2.3	>500	3.6	7	0.5	53	16
visabron *c* (4–4)	>10000	>10000	>10000	0.4	>500	0.4	5	0.4	11	10
visabron *c* (6–6)	>10000	>10000	>10000	0.7	>500	0.9	5	4	20	15
Arg-visabron *c* (4–4)	>10000	>10000	>10000	>10000	nt	>10000	>5000	>5000	nt	nt

a α1- and α2-K562 overexpressed cells, wild-type K562 (α5β1), α9-B1LBC3 overexpressed cells, Jurkat lymphocytes (α4β1), and U937 macrophages (α4β1,αMβ2, α3β1, α6β1), were incubated for 30 min at 37 °C with the cells (1 × 10^5^) in the 96-well plates covered with immobilized (3 mg/mL) extracellular matrix ligand in 100 μL of HBSS containing calcium and magnesium. The inhibitory dose 50% (IC_50_) values representing mean ± standard deviations of three experiments were calculated from the dose–response curves. Abbreviations: VCAM-1, vascular cell adhesion molecule-1; TNFα, tumor necrosis alpha; PMA, phorbol 12-myristate 13-acetate; nt, not tested.

b Experiments performed by ELISA binding assay using recombinant human VCAM-1 and MAdCAM-1 chimera.

c TNF-α (10 ng/mL) treatment for 24 h.

d PMA (10 ng/mL) treatment for 24 h.

### Characterization of Visabron *c* (4–4) as a Leukocyte-Trafficking Inhibitor and as a Therapeutic Agent for EAE

**Figure 2:**
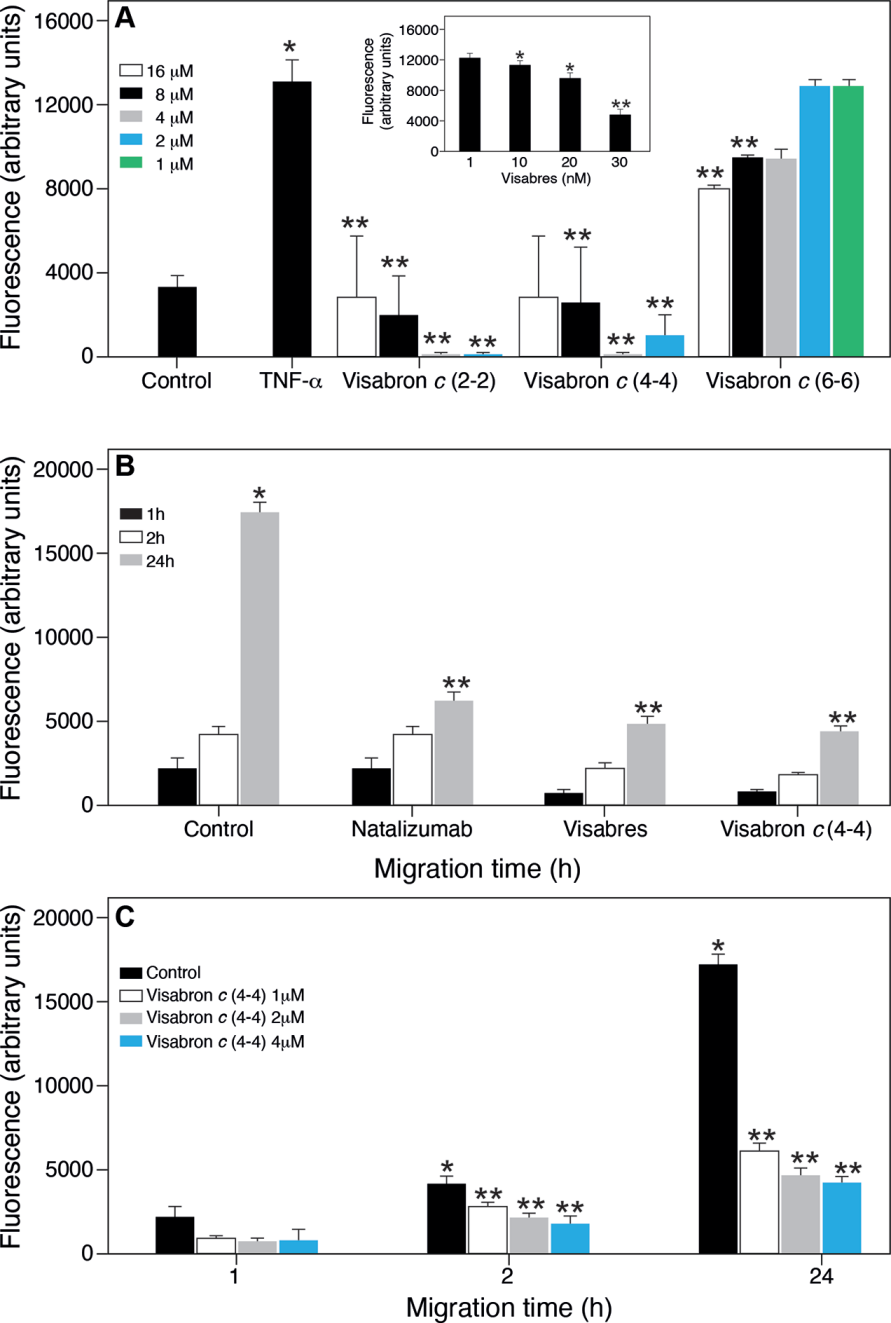
Inhibitory effects of visabron *c* (4–4) on adhesion (A) and transmigration (B, C) of Jurkat cells through the monolayer of TNFα-activated HUVEC. (A) HUVEC were cultured on 96 well plates to 90% confluence in EGM-2 complete media. TNF-α (10 ng/mL) was added to each well for 16 h. Jurkat cells were labeled with calcein-AM (5 mg/mL) by 1 h incubation at 37 °C. After three washes, Jurkat cells (1 × 10^5^ cells per mL) were mixed with appropriate concentrations of visabron or 10 μM visabres (inset) in EGM-2 media without FBS and supplements (growth factors) and incubated for 15 min at room temperature. Thereafter, the Jurkat cells were applied to the wells on the top of the HUVEC monolayer and the plates were incubated 90 min at 37 °C in a CO_2_ incubator. The plates were washed three times with HBSS containing calcium and magnesium, and the cells were solubilized with 1% Triton X-100. Cell cultures extracts were measured using the fluorescence plate reader. Bars represent different concentrations. (B,C) Fluoroblock membranes of the Boyden chambers were coated with fibronectin (2 mg/mL) by incubation in PBS for 1 h at room temperature. HUVEC (5 × 10^5^) was applied on Fluoroblock membranes and incubated overnight at 37 °C in EGM-2 media to completely cover the membrane and to generate the monolayer. The next day, the medium was changed to a fresh medium containing 10 ng/mL of TNF-α, and incubation was continued for an additional 24 h. Jurkat cells were labeled with calcein-AM (10 mg/mL) by incubation at 37 °C for 1 h in RPMI media and after washing, exposed to 1 mg/mL of visabres or natalizumab or visabron *c* (4–4) in the same media containing 1% BSA. Control cells were plated and treated in media only. The suspension of Jurkat cells containing or lacking α4β1 integrin antagonists was applied on the top of the Fluoroblock membrane. The bottom chamber was filled with RPMI/BSA media only. Chambers were incubated at 37 °C, and measurement of fluorescence was performed at the indicated time points using a fluorescence plate reader with a set up bottom measurement option and using FITC filters. Data represent mean ± SD of sixplicate chambers; *p ≤ 0.05, **p ≤ 0.01 compared to respective control.

Leukocytes migrate through vascular endothelium and then into the connective tissue. As an *in vitro* model of this process, we investigated the effect of visabron *c* (4–4) on Jurkat cell adhesion and migration through the human umbilical vein endothelial cell (HUVEC) monolayer activated by the proinflammatory cytokine TNFα. Minimal spontaneous adhesion (8–12%) occurred, but this increased markedly (25–35% of added Jurkat cells) when the cells were activated with TNFα (Figure 2A). The adhesion was strongly inhibited by visabron *c* (2–2) and *c* (4–4) in the range of 1–10 μM (Figure 2A) but was poorly affected by visabron *c* (6–6), while visabres was very active and used as a positive control (Figure 2A, inset). Migration at 24 h across TNFα-stimulated HUVEC was significantly inhibited by 70% upon treatment with visabron *c* (4–4), similar to natalizumab and visabres, indicating its α4β1-mediated, leukocyte-trafficking-inhibitory activity (Figure 2B,C). These findings propose visabron *c* (4–4)’s pharmacological action as a novel leukocyte trafficking inhibitor.

**Figure 3:**
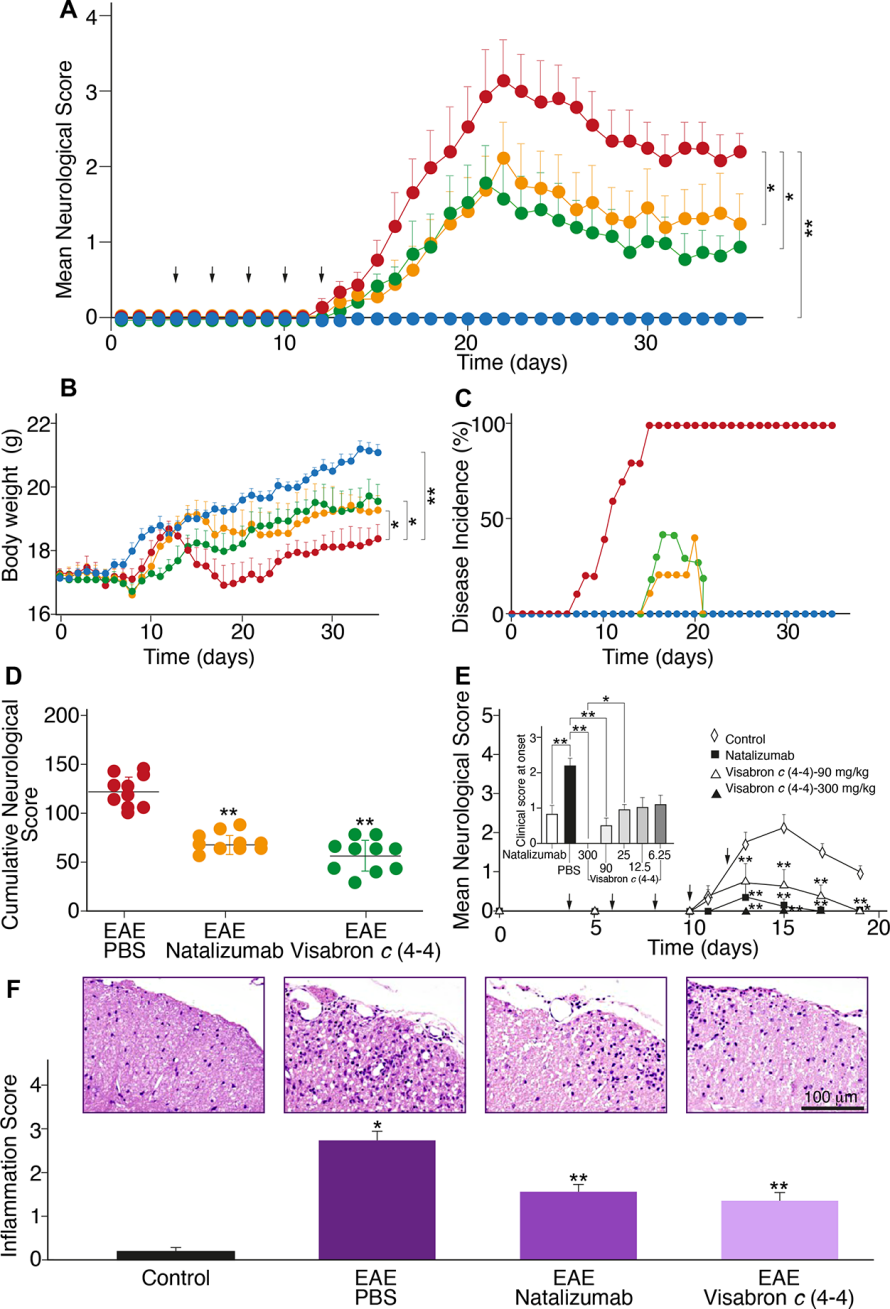
Effect of anti-α4β1 prophylactic treatment with visabron *c* (4–4) compared to natalizumab, on the induction of progressive EAE. EAE was induced in C57bl/6 mice by immunization with MOG/CFA and injection of pertussis toxin as described in the Experimental Section. The body weight, neurological scores, and disease incidence were monitored daily for 35 days. Immunized mice (*n* = 10–14 in each group) were treated (i.p. injection) at time points indicated by arrows. Histology was performed on day 35 on all groups. Data shown are the mean ± SEM, evaluated by the nonparametric Kruskal–Wallis test followed by a Dunn’s postanalysis. All *p*-values <0.05 were considered statistically significant. (A) Neurological clinical score. Visabron *c* (4–4) (green) at a cumulative dose of 25 mg/kg (5 mg/kg every other day, for 8 days) and natalizumab (orange) at a cumulative dose of 120 mg/kg (24 mg/kg every other day, for 8 days). (B) Body weight measurements; *p < 0.05 compared to untreated, EAE disease–mice (red); **p < 0.01 compared to control mice (blue) (C) Disease incidence estimation. (D) Cumulative clinical neurological score of different groups; **p < 0.01 compared to EAE/PBS group. (E) Dose–response of treatment with visabron *c* (4–4). Inset: Mean neurological score of the different treatment groups at disease onset (days 15–17). Mice were randomly allocated into seven treatment groups: 300 (*n* = 4), 90 (*n* = 4), 25 (*n* = 8), 12.5 (*n* = 8), and 6.25 (*n* = 8) mg/kg, PBS (*n* = 11) used as negative control and 120 mg/kg of ntalizumab (*n* = 11), used as positive control. *p < 0.05, for the comparison between PBS and 25 mg/kg of visabron *c* (4–4). **p < 0.01 for the comparison between PBS and either natalizumab or 300 and 90 mg/kg of visabron *c* (4–4). (F) Inflammatory infiltrates (blue) in the spinal cord were evaluated by cell counting in H&E stained slices. Scale bar: 100 μm. *p < 0.01, for comparison to the control and **p < 0.05 for comparison with the EAE/PBS group.

To evaluate the therapeutic effect of visabron *c* (4–4) compared to natalizumab on mice with EAE, the body weight and clinical neurological score were tested daily for up to 35 days. No neurological symptoms were observed during the entire period of 35 days in the nondisease, control group. The EAE disease group began to show neurological deficit symptoms at 13–16 days postimmunization, and the mean neurological score of mice with EAE increased rapidly, reaching the maximal level at 21 days postimmunization. From the onset of disease (13–16 days) to 35 days postimmunization, the increase in the neurological score in the visabron *c* (4–4) and natalizumab groups was significantly lower than that in the EAE group (Figure 3A). The body weights of mice with EAE were markedly decreased from 13 to 16 to 35 days postimmunization, compared with those of control mice; however, this trend was significantly decreased in visabron *c* (4–4) and natalizumab groups (Figure 3B). The lower disease incidence in the visabron *c* (4–4) and natalizumab groups (Figure 3C) and the higher body weight of mice in these groups, in comparison to the EAE disease group, indicate a reduction in disease severity. The cumulative neurological scores of mice at 35 days postimmunization in visabron *c* (4–4) and natalizumab groups were also significantly decreased compared with those of the EAE/PBS group (Figure 3D). Visabron *c* (4–4), in a dose-dependent fashion, induced a significant therapeutic preventive effect in the mice with EAE, similar to natalizumab used as a positive control, in a range of 12.5 mg/kg until 300 mg/kg, as measured by the clinical neurological score at the onset of the disease (Figure 3E). The effect of visabron *c* (4–4) and natalizumab on the degree of inflammatory cell infiltration in the spinal cord tissue was measured by cell counting on H&E stained slices. The EAE-disease group showed a larger amount of inflammatory cell infiltration than the control group. Treatment with either visabron *c* (4–4) or natalizumab significantly reduced the inflammatory cell infiltration (Figure 3F), reflecting their inhibitory effect on α4-integrin-mediated leukocyte trafficking from the blood to the central nervous system. Moreover, since the interaction between α9β1 integrin and tenascin-c modulates the egress of lymphocytes from lymph nodes^26^ and of hematopoietic stem and progenitor cells from bone marrow,^27^ it is tempting to propose that visabron *c* (4–4), being a dual antagonist of α4β1/α9β1 integrins, is also blocking these pathways, enhancing the therapeutic effect on mice with EAE disease.

### Pharmacokinetics of Visabron *c* (4–4)

The cyclic visabrons have high metabolic stability in rat plasma, relative to the linear parent peptides, due to their backbone cyclization (Figure S7). They were stable after 180 min of incubation in rat plasma, while the half-life of the linear visabron was only 7 min, indicating fast degradation of the linear peptide by plasma enzymes. Following i.v. dose administration in rats, visabron *c* (4–4) exposure was with a mean plasma area under the curve (AUC) value of 46.6 + 2 μg·min/mL. The mean clearance (CL) value was 11.08 + 0.5 mL/min/kg, and the volume of distribution was 0.39 + 0.03 L/kg. The mean half-life (*t*_1/2_) was 23.8 + 1.6 min (Table 3). The apparent short elimination half-life indicates that this peptide was rapidly cleared from the plasma by the major eliminating organs the kidneys and the liver. The apparent very small volume of distribution implies that visabron *c* (4–4) has minimal distribution into the tissues, which is in accord with the site of its pharmacologic activity, preventing lymphocyte extravasation from the blood vessels into the extravascular fluids, also indicating that it can undergo efficient elimination by the clearing organs kidney and liver.

**Table 3:** Summary of Mean Pharmacokinetic Parameters in Plasma from Rats Following a Single i.v. Dose Administration of 0.515 mg/kg (*n* = 4) of Visabron *c* (4–4)^a^

pharmacokinetic parameters	unit	value
CL	mL/min/kg	11.08 ± 0.5
*t*_1/2_	min	23.8 ± 1.6
Vd	mL/kg	381 ± 29
AUC	μg·min/mL	46.6 ± 2

a The pharmacokinetic parameters were calculated using noncompartmental analysis with WinNonLin.

Several toxin-derived peptides that have become drugs were used for the management of diabetes, hypertension, chronic pain, and other medical conditions.^14^ Despite the similarity in their amino acid composition, toxin-derived peptide drugs have very profound differences in their structure and conformation, in their physicochemical properties (that affect solubility, stability, etc.), and subsequently in their pharmacokinetics.^28^

As can be seen from Figures S8 and S9, the relationship between the PK profile of visabron *c* (4–4) and its pharmacodynamics therapeutic effects are indirect,^29^ taking time for development, and are not apparently related to plasma concentration, reminiscent of trafficking behavior of helper T cells in response to methylprednisolone.^30^ In the EAE model experiment, visabron *c* (4–4) was administered i.p. every 48 h by five injections. Given that the peptide’s half-life is 24 min, it is assumed that following 4 half-lives (approximately 96 min), visabron *c* (4–4) will be eliminated from the plasma. Therefore, it can be concluded that there is an “indirect pharmacodynamic (PD)” relationship since >48 h postadministration in the EAE experiment the active peptide visabron *c* (4–4) no longer exists in the plasma, and yet significant therapeutic effects were exhibited among mice treated with visabron *c* (4–4) at cumulative doses of 300, 90, and 25 mg/kg. Therefore, the PK/PD relationship of this cyclic peptide is complex and is a type of “indirect PD” and is predicted to be depended on various biological parameters such as expression and activation levels of the α4β1/α9β1 integrins, lymphocytes turnover, and their trafficking regulation and not solely by the plasma concentration level of visabron *c* (4–4). To highlight this indirect PD phenomenon it is important to note that the kinetics of the measured (clinical/therapeutic) response is much slower than the elimination kinetics of the active cyclic peptide visabron *c* (4–4) in the body.

### Safety of Visabron *c* (4–4)

**Figure 4:**
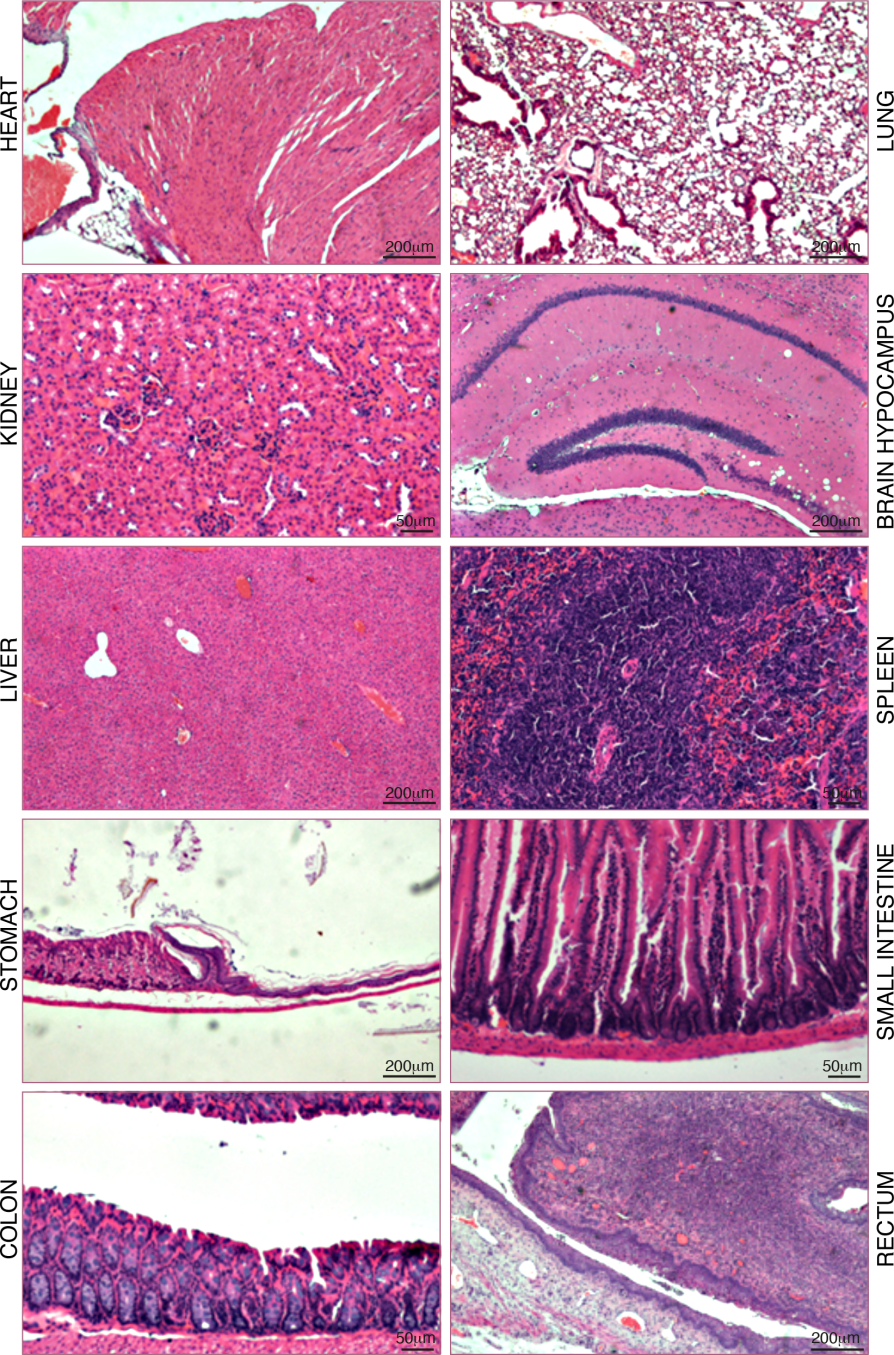
Representative histological images of organ slices stained with hematoxylin and eosin (H&E) for acute toxicity test of male mice 48 h after i.v. administration of 500 mg/kg of visabron *c* (4–4).

**Figure 5:**
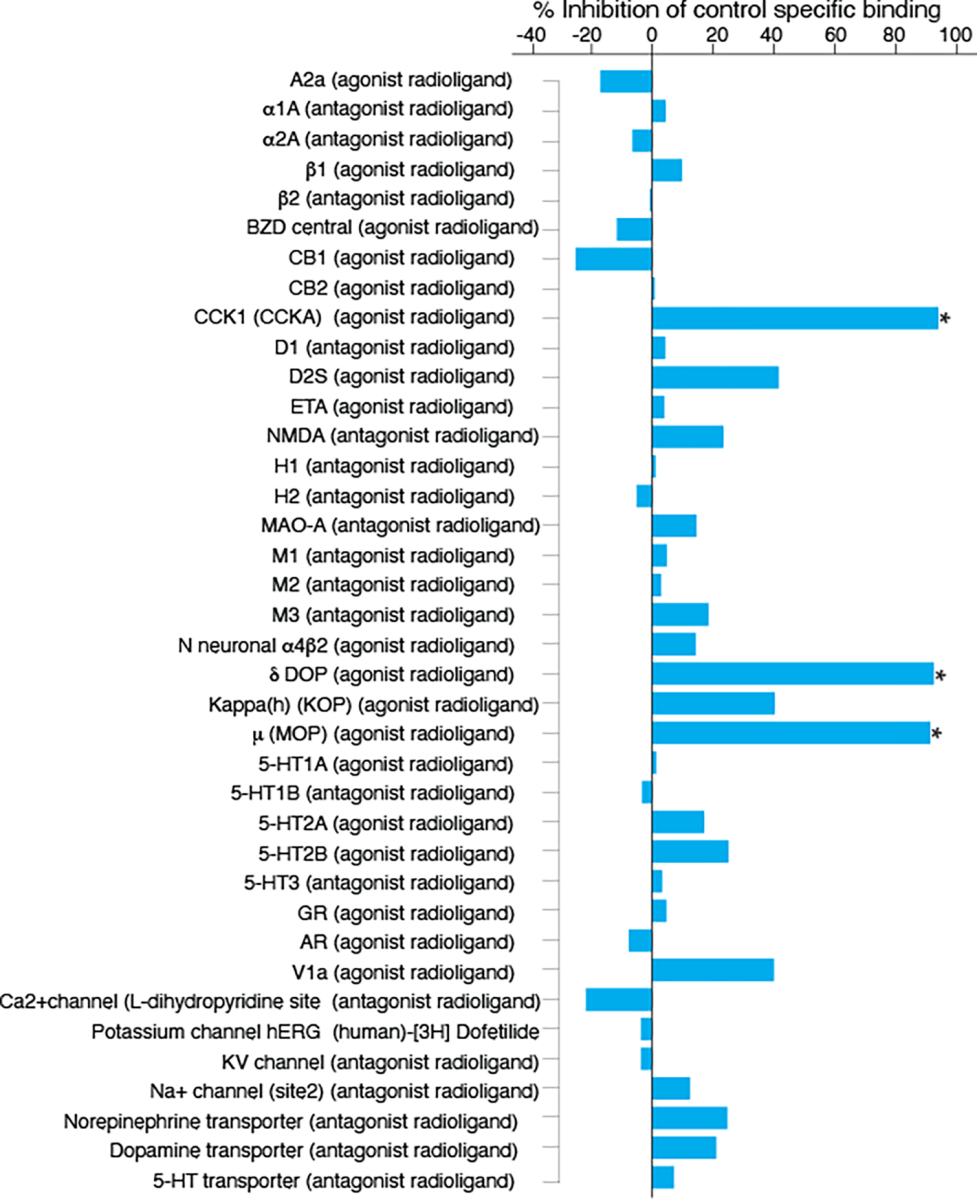
Screening of potentially significant off-target effects of visabron *c* (4–4) performed via SafetyScreen44. The data expressed as mean percentage of inhibition of control-specific binding of a radioactively labeled ligand specific for each target, represent visabron c (4–4) tested at 1 mM. 100 μM visabron *c* (4–4) was without any significant effect (less than 20%) on drug-specific binding to all targets. Results showing inhibition higher than 50% are considered to represent significant effects. Graphs are presented as the mean of duplicate assays.

**Figure 6:**
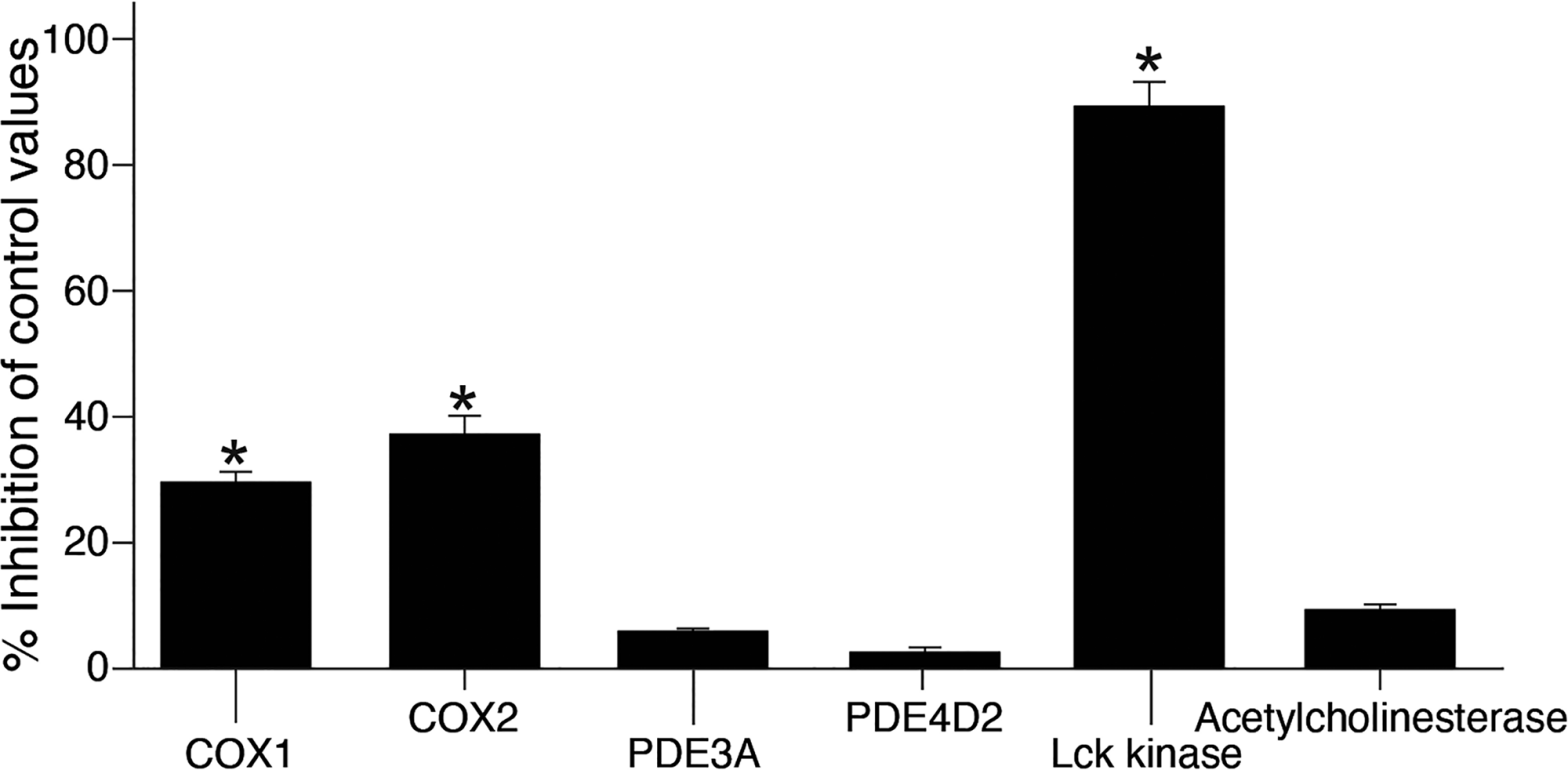
In vitro enzyme inhibition by 1 mM visabron *c* (4–4). Results show mean percentage of control enzyme activity inhibition, and values higher than 50% are considered to represent significant effects. Graphs present the mean of duplicate assays.

Single-dose toxicity evaluation by hematocrit cell counting and biochemical analysis indicated acute tolerability of 500 mg/kg of visabron *c* (4–4) 24 h after i.v. injection in C57BL/6 male mice (Tables S9 and S10), without short-term adverse pathological effects on major organs, when administered either i.v. or per o.s., at a dose of 500 mg/kg for a period of 48 h (Figure 4 ;Table S11). The “off-target” effect may result in adverse effects of the drug resulting in drug withdrawal. Therefore, *in vitro* pharmacological profiling is increasingly being used earlier in the drug discovery process to identify undesirable off-target activity profiles that could hinder or halt the development of a candidate drug.^31^ Eurofins Cerep Safety scan *in vitro* analyses of visabron *c* (4–4), including 44 binding, enzymatic, and transporters panel assays, provided data regarding the potential radioligand binding competition by visabron *c* (4–4) on major physiological targets, known to show a clear correlation with observed *in vivo* toxic effects.^31^ We chose to investigate a concentration of 1 mM visabron *c* (4–4) which is about 200-fold higher than the IC_50_ for α4β1 integrin inhibition using “in cell” assay and assumed to be higher than the therapeutic window, in order to predict visabron *c* (4–4) interactions at toxic overdoses. We set a threshold of 40% for binding competition on all tested targets as summarized in Figure 5. The results indicated that visabron *c* (4–4) did not interact with the majority of the physiological targets, such as (a) cholinergic muscarinic receptors; (b) α and β adrenergic receptors subtypes; (c) cannabinoid receptors; (d) GABA (benzodiazepine) receptors; (e) several dopaminergic and (f) serotonergic receptors; (g) glutamatergic and (h) histaminergic receptors; (i) glucocorticoid and (j) androgen receptors; (k) vasopressin receptor, and the most abundant (l) voltage-dependent sodium, potassium, and calcium channels, and (m) the typical catecholamine transporters for norepinephrine, dopamine, and serotonin. However, at this high concentration, but not at 100 μM, visabron *c* (4–4) competed with radioligand agonists of three G-protein coupled pain receptors (GPCRs), cholecystokinin CCK1 (CCKA) (94.4%) and opiate receptors δ (DOP) (93%) and μ (MOP) (92%) (Figure 5), and moderately inhibited cyclooxygenase (COX) enzymes and strongly inhibited Lck kinase activity (Figure 6). However, this inhibitory effect of Lck was not observed using 100 μM visabron *c* (4–4) (data not shown). Cumulatively, these findings support the high selectivity of visabron *c* (4–4) on an α4β1/α9β1 integrin target and indicate that at a very high concentration it may cause analgesic effects by binding to opiate receptors and inhibiting cyclooxygenases, adverse effects that may be beneficial in multiple sclerosis patients that complain of pain. Safety screening in early drug discovery tested compounds at a 10 μM concentration and assume that a high number of off-target hits (up to 14) generally led to severe side effects, which later led to *in vivo* screening only occurring with molecules having <7 off-targets.^32^ Application of this safety lead optimization screening strategy during the present early stage of drug discovery led to the identification of visabron *c* (4–4) with CCK and opioid receptors off-target binding at a very high, nonphysiological concentration. In the case of CCK1 there is sequence similarity between visabron *c* (4–4) and CCK1^33^ as can be seen in Figure S10, which can account for the binding of visabron *c* (4–4) to the CCK receptors. In the case of the opioids, visabron *c* (4–4) did not possess the main pharmacophore groups, namely two aromatic rings, an aromatic hydroxyl group, and a polar or charged nitrogen essential for binding to the opioid MOP and DOP receptors.^34^ Visabron *c* (4–4) contains two out of four pharmacophores essential for opioid receptor binding and activation (NH_3_^+^ of Lys^39^ and OH of Thr^41^); however, it does not contain the two aromatic rings unless we consider the side chains of Met^42^, Leu^43^, and Leu^46^ as aromatic isosteres.^35^ It may be hypothesized that at a very high concentration, Visabron *c* (4–4) will be a positive allosteric modulator of the opioid receptors, similar to several nonpeptidyl small molecules.^36^ However, since at 100 μM visabron *c* (4–4) did not interact with any of the above receptors, it is less plausible that *in vivo* visabron *c* (4–4) will activate opioid receptors. Nevertheless, to ensure risk mitigation, this possibility needs to be confirmed by future experiments to get a full pharmacological profile of visabron *c* (4–4)’s effect on CCK and opioid receptors to safely exclude the possibility of potential side effects.

**Figure 7:**
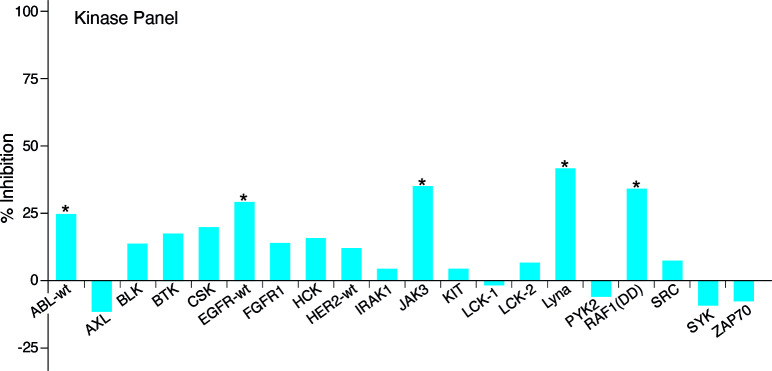
Testing the potency of visabron *c* (4–4) on a panel of leukocyte tyrosine protein kinases using the PhosphoSens technology_._ The data is presented as mean percentage inhibition of duplicate assays.

To further characterize the leukocyte target selectivity, the effect of visabron *c* (4–4) was investigated on a panel of 20 tyrosine protein kinases of immune cells that were reported to be involved in leukocyte trafficking, immunosuppression, and/or induction of inflammation.^37,38^ The measurements of the activity of cytoplasmic and receptor recombinant tyrosine protein kinases were based on the level of chelation-enhanced fluorescence that is directly proportional to the amount of phosphorylated, real-time sensors consisting of sulfonamido-oxine (Sox) chromophore linked to a peptide or protein substrate.^39^ At a concentration of 1 mM, but not 0.1 mM, visabron *c* (4–4) inhibited from 25% to 40% Abl, EGFR, JAK3, LynA, and C-Raf (Figure 7), indicating that this cyclic peptide did not target leukocyte tyrosine protein kinases that regulate the production of inflammatory mediators at a concentration a hundred-fold higher than the IC_50_ for inhibition of α4β1 integrin. From a mechanistic point of view, these data strongly propose that the leukocyte trafficking inhibition induced by low micromolar concentrations of visabron *c* (4–4) (Figure 2) was solely determined by α4β1 integrin antagonism but not by immunosuppression mediated by inhibition of a putative leukocyte’ tyrosine protein kinase.

**Figure 8:**
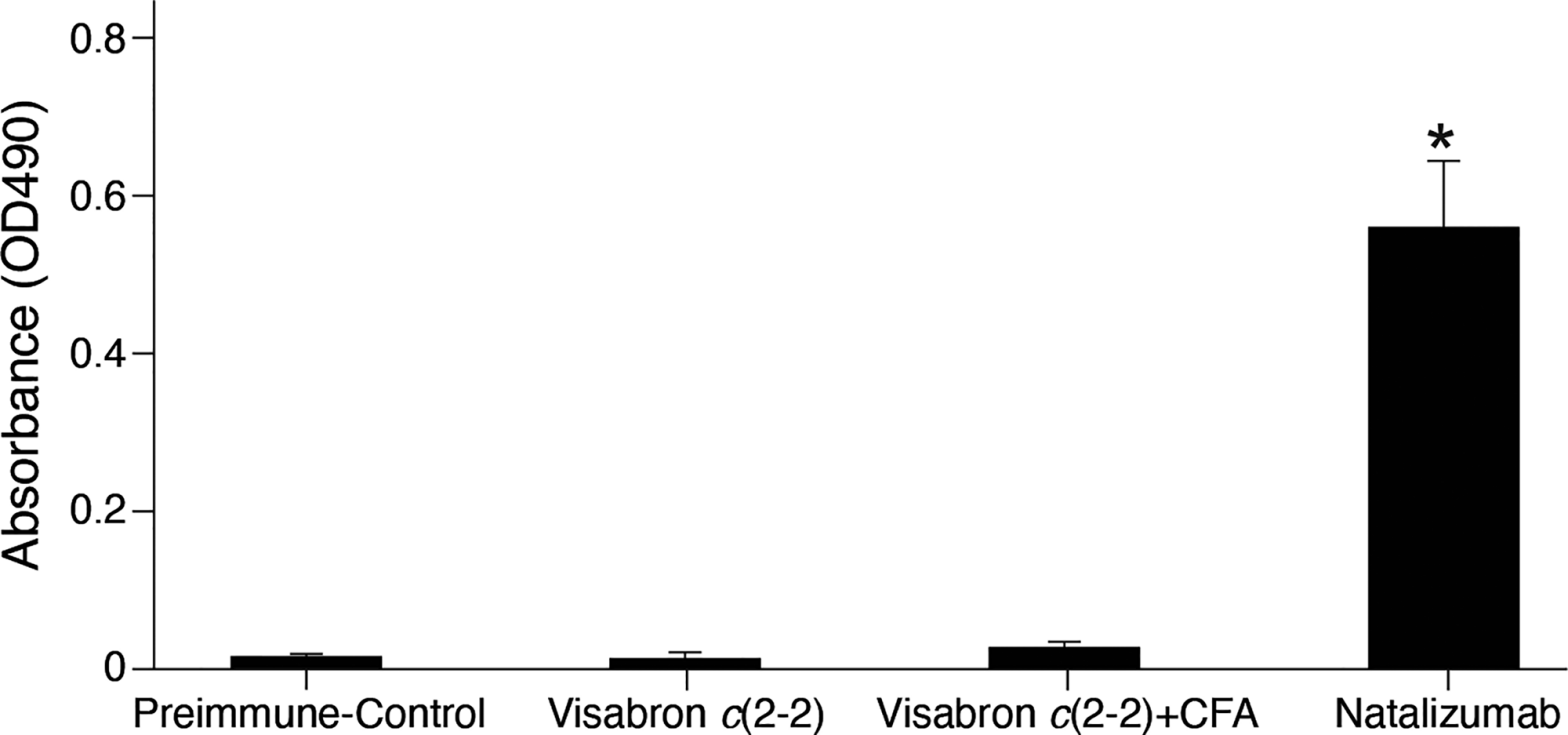
Antibody titer test in BALB/c mice, 40 days post intraperitoneal administration with natalizumab (18 mg/kg), visabron *c* (2–2) (150 mg/kg) alone, and administered together with Complete Freund’s Adjuvant (visabron *c* (2–2) + CFA), as compared to preimmune control serum. The results represent the mean ± SEM (*n* = 6) of optical absorbance (OD 490 nm) in ELISA. *p ≤ 0.01 compared to control.

Natalizumab, being a large protein, is immunogenic, especially when used as monotherapy, inducing in 6–9% of multiple sclerosis patients anti-natalizumab-neutralizing antibodies and relapses that occurred by therapy inhibition.^40^ In contrast to natalizumab, visabron *c* (2–2) alone or together with Freund‘s complete adjuvant did not induce antibody production in a BALB/c mice model, as evident from the lack of a significant reaction in ELISA by comparison to the preimmune serum (Figure 8). This finding was also supported by the very low immunogenicity score of a few percentages for the visabron sequence, calculated using the NHLBI-ABDesigner tool.^41^

In conclusion, the use of backbone cyclization to constrain the TMLD peptide motif in defined conformational space has led to the selection of visabron *c* (4–4). Visabron *c* (4–4) was synthesized, characterized, and pharmacologically identified as a selective backbone cyclic peptide antagonist of VCAM-1/ α4β1/α9β1 integrins, blocking leukocyte migration *in vitro,* and conferring therapy in MS-EAE *in vivo* mice model. Visabron *c* (4–4) is proposed as an alternative backbone cyclic peptide drug to monoclonal anti-α4 integrin due to its safety properties, lack of immunogenicity, and low price of production. Visabron *c* (4–4) can be delivered systemically and represent a safe alternative to steroids and immunosuppressant drugs for therapy of autoimmune inflammatory diseases. We are currently conducting structural modifications of Visabron *c* (4–4) to generate a prodrug for oral application. Additional preclinical *ex vivo* and *in vivo* experimentation is necessary to confirm whether visabron *c* (4–4) possesses a full safety liability before conducting first-in-human studies.

## Experimental Section

### Materials

#### Chemicals

1-[Bis(dimethylamino)methylene]-1*H*-1,2,3-triazolo[4,5-*b*]pyridinium-3-oxide hexafluorophosphate (HATU), 1-hydroxy-7-azabenzotriazole (HOAt), 9-fluorenylmethyloxycarbonyl-*N*^α^-protected amino acids (Fmoc-N^α^-AA-OH), and *N*-(9-fluorenylmethoxycarbonyloxy)succinimide (Fmoc-OSu) were purchased form Chem-Impex International, Inc. (Wood Dale, IL). Fmoc-Rink-amide methylbenzhydrylamine (MBHA) resin (200–400 mesh, 0.66 mmol/g resin) was purchased from Iris Biotech GmbH (Marktredwitz, Germany). 1,2-Diaminoethane, 1,4-diaminobutane, 1,6-diaminohexane, 2,4,6-trimethylpyridine (collidine), bis(trichloromethyl) carbonate (BTC), tetrakis(triphenylphosphine) palladium(0), diethyl ether, bromoacetic acid, acetic anhydride (Ac_2_O), piperidine, trifluoroacetic acid (TFA), diisopropylethylamine (DIPEA), methanol (MeOH), triethylamine (Et_3_N), triisopropylsilane (TIS), dibromomethane (DBM), dimethyl sulfoxide (DMSO), and other organic materials were purchased from Across Organics N.V. (Geel, Belgium). Organic solvents for solid-phase peptide synthesis (SPPS) and for high-performance liquid chromatography (HPLC) including *N*-methyl-2-pyrrolidone (NMP), dichloromethane (DCM), *N*,*N*-dimethylformamide (DMF), and acetonitrile (ACN) were purchased from J.T. Baker (NJ). CellTracker Green 5-chloromethylfluorescein diacetate (CMFDA) was purchased from Invitrogen/Molecular Probes (Eugene, ORA). Human recombinant tumor necrosis factor-α (TNFα), phorbol 12-myristate 13-acetate (PMA), and Complete Freund’s Adjuvant were purchased from Sigma (Rehovot, Israel). Other chemicals and reagents were of analytical grade. Pertussis toxin was obtained from List Biological Laboratories, Campbell, CA.

#### Integrins and Ligands

Collagen IV (from bovine placenta villi) was purchased from Chemicon Temecula, CA, and Collagen I (from rat tail) was purchased from BD Biosciences (Bedford, MA). Recombinant Human VCAM-1 was purchased from PeproTech Co. (Rehovot, Israel), and recombinant human VCAM-1/CD106 Fc and MAdCAM-1 Fc chimeras, as well as recombinant human integrin α4β1 or α4β7 were obtained from R&D Systems Co (Minneapolis, MN). Human fibronectin and laminin were purchased from Sigma-Aldrich Israel Ltd. (Rehovot, Israel).

#### Monoclonal Antibodies and Disintegrins

The clinical-grade monoclonal antibodies natalyzumab (Tysabri) anti-α4β1 produced by Biogen Co. (Cambridge, MA) and vedolizumab (Entyvio) anti-α4β7, produced by Takeda Co. (Tokyo, Japan), were a kind gift from the pharmacy of the Hadassah Hebrew University Medical Center. The anti-integrin α9β1 monoclonal antibody [Y9A2] was purchased from Abcam Co. Cambridge, UK (ab27947), and the polyclonal and monoclonal antibodies against α4β1 and α4β7 were purchased from LifeSpan BioSciences Inc. (Seattle, WA) and ThermoFischer Scientific Co. (Waltham, MA), respectively. The anti-α4 disintegrin VLO5 was purified from the venom of *Vipera lebetina* obtuse.^42^ The disintegrin anti-α1β1 viperistatin and the C-type lectin protein anti-α2β1 vixapatin were isolated from the venom of *Vipera xantina palestinae* as previously described.^43,44^

#### Snake Venoms

The venom of *V. lebetina obtusa* was purchased from Latoxan Serpentarium (Valence, France). The venom of the Israeli *Vipera daboia* was purchased from SIS Co. (Rehovot, Israel) which collected and maintained the snakes under good laboratory practice (GLP) conditions, according to the requirements of the Israeli Ministry of Health for antiserum production.

#### Peptide Synthesis

All of the reactions that were performed on solid support utilized Fmoc chemistry for the Nα protection. The reactions were shaken using a Bigger Bill orbital shaker. Preactivation tubes were stirred over a vortex. Excluding the cleavage procedure, all other reactions on the solid support were performed under basic conditions of pH 8–9. The process of the reactions was monitored by HPLC/MS following a “small cleavage” procedure. The synthesis via solid support was performed in a vessel equipped with a sintered glass bottom. Fmoc Rink Amide methylbenzhydrylamine (Fmoc Rink Amide-MBHA) resin (loading capacity 0.66 mmol/g resin) was the solid support for this synthesis. The equivalents of all reagents used in SPPS were calculated with respect to the resin loading capacity and weight. The volume of the solvents in all of the reactions was fixed to 30 mL to maintain a constant concentration of reagents. After cleavage from the resin, the crude final peptides were dissolved in TDW/ACN 1:1 mixture, lyophilized, and analyzed by HPLC/MS.

#### Coupling Protocol

A solution of Fmoc-N^α^-AA-OH (3 equiv) and HOAt (3 equiv) in NMP was prepared and cooled to 0 °C. HATU (3 equiv) was then added for preactivation of the amino acid prior to reaction with the peptidyl-resin, and the solution was shaken at 0 °C for 3 min. The preactivated solution was added to the peptidyl-resin, and the mixture was shaken for 60 min. The procedure was repeated twice. The resin beads were washed with 30 mL NMP (4 × 2 min) and DCM (2 × 2 min).

#### Fmoc Removal

For the removal of the Fmoc protecting group, a solution of 20% piperidine in NMP was added to the peptidyl-resin. The reaction was performed in a vessel shaken at room temperature for 30 min. The reaction was repeated once using a fresh solution of 20% piperidine in NMP. At the end of the second cycle, the peptidyl-resin beads were washed with 30 mL of NMP (4 × 2 min) and DCM (2 × 2 min).

#### Alloc Removal

For the removal of the Alloc protecting group, the peptidyl-resin was added to a saturated argon solution composed of acetic acid (5%), *N*-methylmorpholine (2.5%), and DCM (92.5%), and the mixture was shaken for 5 min, after which tetrakis(triphenylphosphine)palladium(0) Pd(PPh3)4 (0.1 equiv) was added. The reaction mixture was shaken vigorously in the dark and under argon for 3 h, after which the peptidyl-resin beads were washed with a 30 mL solution of 0.5% v/v DIPEA in DMF (5 × 2 min), 0.5% sodium diethyldithiocarbamate trihydrate salt in DMF (5 × 2 min), and DCM (5 × 2 min).

#### Urea Cyclization

On-resin urea cyclization was carried out by adding a solution of BTC (0.33 equiv) and DIPEA (10 equiv) in DCM to the peptidyl-resin and the mixture was shaken for 2 h, after which the resin beads were washed with 30 mL of DCM (4 × 2 min), and DMF (2 × 2 min).

#### Cleavage Protocol

In this method, simultaneous removal of the synthesized peptides from the solid support together with the removal of the acid-labile, side chain protecting groups was performed by the following procedure: 30 mL of precooled solution (at 0 °C) composed of TFA (95%), TDW (2.5%), and TIS (2.5%) was added to dried and desiccated 2.1 g peptide-resin beads. The reaction mixture was kept standing at 0 °C for 30 min after which it was shaken for 150 min at room temperature. The TFA solution containing the cleaved peptide was then separated from the resin beads via filtration, and the TFA was partially evaporated by a stream of nitrogen. Cold diethyl ether was added to the remaining volume of TFA, and the mixture was centrifuged to separate scavengers and other hydrophobic impurities from the precipitated peptide. Diethyl ether was then removed from the precipitate by decanting. The cycle of precipitation, centrifugation, and decanting was repeated three times. The precipitate was dissolved in 10 mL of ACN/TDW (1:1) (relative to 2.1 g peptide-resin beads), and the solution was lyophilized overnight prior to purification via preparative HPLC. The lyophilized crude product was obtained as fluffy white solid.

#### Preparative High-Performance Liquid Chromatography (HPLC)

The crude peptides were dissolved in a TDW/ACN 1:1 mixture, filtered through a 0.45 μm PTFE filters, and injected in 5–10 mL volumes to a reversed-phase preparative HPLC column of Vydac (C18, 22 × 250 mm, 10 μm). The analysis utilized a Merck-Hitachi L-6200A pump and L-7400 variable wavelength detector recording at 220 nm at room temperature. The gradient of the mobile phase consisted of A: TDW (0.1% v/v TFA) and B: ACN (0.085% v/v TFA). First, the column was equilibrated for 5 min at 95% A, and then a linear gradient was applied from 5 to 40 min to reach 95% B. The mobile phase remained for 5 min at 95% B for column equilibration. The gradient was returned back to the starting conditions (95% A, 5% B) within 5 min and kept at this point for additional 5 min for column equilibration. The flow rate of the mobile phase was 9 mL/min. The collected fractions were analyzed by MS and lyophilized, and samples were injected into an analytical HPLC column to determine the degree of purity.

#### Analytical HPLC

All samples were dissolved in a TDW/ACN 1:1 mixture, filtered through a 0.45 μm PTFE filter, and injected into a reversed-phase analytical HPLC column of Vydac (C18, 4.6 × 250 mm, 10 μm). The analysis utilized a Merck-Hitachi L-7100 pump and L-7400 variable wavelength detector recording at 220 nm at room temperature. The gradient of the mobile phase consisted of (A) TDW (0.1% v/v TFA) and (B) ACN (0.085% v/v TFA). First, the column was equilibrated for 5 min at 95% A, and then a linear gradient was applied from 5 to 20 min to reach 95% B. The mobile phase remained for 5 min at 95% B for column equilibration. The gradient was returned back to the starting conditions (95% A, 5% B) within 5 min and kept at this point for additional 5 min for column equilibration. The flow rate of the mobile phase was 1 mL/min. The collected fractions were further analyzed by MS.

#### Mass Spectrometry (MS)

Mass spectra were acquired on a LCQ Fleet Ion Trap mass spectrometer (Thermo Scientific) utilizing electrospray ionization. For HRMS analyses, the spectra were recorded on an Agilent 6550 iFunnel Q-TOF LC/MS system.

#### Solid-Phase Ligand–receptor Binding

Recombinant human VCAM-1 and MadCAM-1 (3 mg/mL in phosphate-buffered saline containing divalent cations), were immobilized on the wells of a 96-well ELISA plate (Dynatech Immulon) by overnight incubation at 4 °C. After washing with HBSST (Hanks’ Balanced Salt solution containing calcium and magnesium and 0.05% Tween-20) the plates were blocked with 5% BSA in HBSST by incubation at 37 °C for 1 h. The plates were washed with HBSST, and recombinant human integrin α4β1 or α4β7 (3 mg/mL) was added in the presence or absence of competitor cyclic peptides, disintegrins, or monoclonal anti-integrin antibodies dissolved in HBSS containing 1% BSA. The plates were thereafter incubated at 30 °C for 3 h, as time-binding had reached a plateau, and then washed 3 times with HBSST. Bound integrins were quantitated using primary anti-integrin polyclonal and monoclonal antibodies at a concentration of 0.5 mg/mL in HBSST containing 1% BSA, and incubated at 37 °C for 1 h. The plates were washed 3 times with HBSST and secondary antibodies- alkaline phosphatase (AP) conjugated (Sigma Co.) were added at dilution 1:1000 and incubated at 37 °C for 1 h. Thereafter, the plates were washed 3 times with HBSST and twice with HBSS and the alkaline phosphatase substrate 4-nitrophenyl phosphate was added to the wells for 30 min. The plates were read using Tecan Spectrophotometer at 405 nm. The level of nonspecific binding was measured in each experiment by determining the level of binding to wells coated with BSA alone. Control experiments indicated that the interaction of ligands to integrins was specific, since the binding was significantly and equally, inhibited by EDTA, selective disintegrins, and by antifunctional monoclonal antibodies directed against either the α4β1 or α4β7.

#### Cell Lines and Culture Conditions

K562 cells transfected with α1and α2 integrins were originally provided by Dr. M. Hemler (Dana Farber Cancer Institute, Boston, MA). Κ562 human immortalized myelogenous leukemia cell line, Jurkat immortalized human T-lymphocyte cell line, Ramos (RA1, B-lymphocytes lymphoma cell line), SW480 epithelial human adenocarcinoma cell line, and U-937 cells with monocytic-type phenotype that differentiate to macrophages following PMA stimulation were purchased from ATCC (Manassas, VA). Human umbilical vein endothelial (HUVE) cells were purchased from Clonetics (San Diego, CA), grown in endothelial cell growth medium containing 2% fetal bovine serum, human recombinant epidermal growth factor (10 ng/mL), gentamycin (50 mg/mL), amphotericin B (50 ng/mL), bovine brain extract (12 mg/mL), and hydrocortisone (1 mg/mL), and used between passage 4 and 8. α4 and α9- and mock-transfected SW480 and glioma cells were generated by transfection with full-length α9 expression plasmid pcDNAIneoa9^45,46^ or with the empty vector pcDNAneoI (InVitrogen, San Diego, CA) by calcium phosphate precipitation. Transfected cells were maintained in Dulbecco’s modified Eagle’s medium (DMEM) supplemented with 10% fetal calf serum and the neomycin analog G-418 (1 mg/mL) (Life Technologies, Inc.). The cell lines expressed high surface levels of integrins as determined by flow cytometry using the respective monoclonal antibodies.

#### Cell Adhesion Assay

Cell adhesion assay was carried out as previously described^47^ with minor modifications. The day before the experiment, each well was coated with VCAM-1 (3 mg/mL) immobilized on the 96-well plate in PBS by overnight incubation at 4 °C. Thereafter, nonspecific binding was blocked by incubating the wells with 1% (w/v) bovine serum albumin (BSA) in Hank’s Balanced Salt Solution (HBSS) containing 5 mM MgCl_2_ at room temperature for 1 h before use. The cells were labeled by incubation with 12.5 μM 5-chloromethylfluorescein diacetate (CMFDA) in HBSS without 1% BSA at 37 °C for 30 min. The labeled cells were then centrifuged at 1000 rpm and washed twice with HBSS containing 1% BSA to remove excess CMFDA. Labeled cells (about 1 × 10^5^ cells/well) were plated on each well in the presence or absence of visabrons and incubated at 37 °C for 60 min. Unbound cells were removed by washing the wells three times with 1% (w/v) BSA in HBSS, and bound cells were lysed by the addition of 0.5% Triton X-100 (diluted in DDW). The fluorescence in each well was quantified with a SPECTRAFluor Plus plate reader (Tecan), at λ_ex_ = 485 nm and λ_em_ = 530 nm. To determine the number of adhered cells from the fluorescence values, a standard curve was generated by serial dilution of known numbers of CMFDA-labeled cells.

#### Transmigration Assays

*trans*-Endothelial leukocyte migration was assessed as follows: HUVEC cells were plated onto collagen type IV (5 μg/mL) coated polycarbonate inserts (Transwell, Costar, Cambridge, MA; diameter, 6.5 mm; pore size, 8 μm for a 24-well plate) in serum-containing endothelial cell growth medium and allowed to grow to confluence over 72 h. Twenty-four hours before assays, the upper chambers were washed twice with serum-free medium, and a new medium with or without 3 ng/mL TNFα was added. Immediately prior to the addition of the cells, the upper chambers were washed twice with serum-free DMEM, and the medium in the lower chamber was replaced with 500 μL of serum-free DMEM. Jurkat cells labeled with CMFDA for 1 h. Thereafter, the cells were suspended in DMEM at a density of 5 × 10^4^ cells in a volume of 300 μL, preincubated in the presence or absence of visabrons for 1 h at room temperature, and applied on the layer of HUVEC. Insets were placed into a 24-well plate containing 700 μL of DMEM with 2% FBS used as a chemoattractant. After 1, 2, and 24 h at 37 °C in 5% CO_2_, nonadherent cells in the upper chamber were removed. Medium including migrating cells from the lower chamber was collected, the lower chamber was rinsed several times to collect all of the Jurkat cells that had transmigrated, and the absence of additional adherent cells was confirmed microscopically. The medium and all washes were pooled, and the fluorescence in each well was quantified with a SPECTRAFluor Plus plate reader at λ_ex_ = 485 nm and λ_em_ = 530 nm. To determine the number of adhered cells from the fluorescence values, a standard curve was generated, by serial dilution of known numbers of CMFDA-labeled cells. The experiments were carried out in sixplicate and repeated three times.

#### MOG-Induced EAE in C57BL/6 Mice

All animal experiments were conducted under the guidelines and supervision of the Hebrew University Ethical Committee, which approved the methods employed in this project (Permit No. MD-18-15643-5). C57Bl/6 female mice, 8 weeks old, obtained from the Harlan animal breeding center were used in the study and were maintained in the SPF unit of the Faculty of Medicine. EAE was induced by s.c. injection of 0.2 mL/mouse emulsion containing 200 μg of MOG_35–55_ peptide in saline and an equal volume of 5 mg/mL Complete Freund’s Adjuvant (CFA) containing 1 mg/mL heat-killed *M. tuberculosis* (H37Ra; Difco Laboratories, Detroit, MI) . The emulsification was made from equal parts of oil and liquid portions (1:1) in two syringes connected to each other with Leur lock, transferred to an insulin syringe, and 0.2 mL was injected to the right flank of each mouse. On the day of immunization, pertussis toxin (200 ng/mL) was injected i.p. at a volume dose of 0.2 mL/mouse. The injection of the pertussis toxin (i.p) was repeated after 48 h to boost the immune recognition reaction. From day four, natalizumab and Visabron *c* (4–4) were injected i.p. each other day, five times, and the weight, the neurological score and the disease incidence of the mice were monitored until 35 days after immunization. EAE scoring system: mice were observed daily for the appearance of neurological symptoms, which were scored as follows: 0, asymptomatic; 1, partial loss of tail tonicity; 1.5, limp tail; 2, hind limb weakness (right reflex); 3, ataxia; 4, early paralysis; 5, full paralysis; and 6, moribund or dead. The disease incidence was calculated as a percentage of the ratio of the number of new diseased mice per number of living mice at each time-point. In experiments to estimate cell infiltration in the spinal cord at 35 days postimmunization, the mice were euthanized by cervical dislocation and lumbar spinal cords were collected. The mice were perfused with normal saline and 4% paraformaldehyde through the blood circulation and the spinal cord was fixed with 4% paraformaldehyde for 24 h, embedded in paraffin, and cut into 5 μm thick sections. Hematoxylin-Eosin staining (H&E) was performed. To assess the degree of inflammatory cell infiltration, in each mouse, three histological sections were examined. The inflammation score was calculated based on the degree of infiltration of the inflammatory cells: 0, no infiltrating cells; 1, a small amount of infiltrating cells; 2, inflammatory infiltrating tissue around blood vessels; 3, extensive perivascular scar infiltration.

#### Immunogenicity Protocol

BALB/c male mice, aged 60 days (22 gr) were divided into three groups each containing six mice. The mice were immunized intraperitoneal with 0.5 mL of Visabron *c* (2–2) (150 mg/kg), 0.25 mL Visabron *c* (2–2) (150 mg/kg) + 0.25 mL of Freund‘s complete adjuvant, and 0.25 mL natalizumab (18 mg/kg). After 14 days of the first inoculation, the mice received a booster with 0.5 mL of the preparations mentioned above. To verify the presence of antibodies against visabron *c* (2–2) or natalizumab, the serum collected from mice tail blood was tested 40 days after the first inoculation by indirect ELISA using visabron *c* (2–2) or natalizumab antigens immobilized to the plate (25 μg per well, diluted in 20 mM carbonate buffer, pH 9.6 overnight incubated at 4 °C). Then the plates were washed three times with PBS containing 0.05% Tween 20 (T20) and incubated for 1 h at 37 °C with 5% nonfat dry milk in PBS-T20 (blocking buffer, BB). Serum samples (*n* = 18) were diluted at 1:100 in BB, added to the plate, and incubated for 1 h at 37 °C. The plates were then washed three times with PBS–T20. Antimouse whole molecule IgG-peroxidase conjugated (Sigma) was added at a dilution of 1:1500 in BB, and incubated for 1 h at 37 °C. After three washes with PBS–T20, substrate and chromogenic reagent (5 μL H_2_O_2_ and 3.4 mg σ-phenylenediamine in 0.1 M citrate–phosphate buffer, pH 5.0) were added and incubated for 5 min. The reaction was stopped with 12.5% H_2_SO_4_, and absorbance was measured at 490 nm. The results were considered significant when the absorbance was at least two times higher than that obtained with the preimmune serum.

#### Pharmacokinetics

Pharmacokinetics was performed as detailed in the Supporting Information.

#### Pathological Analysis of Major Organs

Eight-week-old C57Bl/6 mice were obtained from Envigo animal breeding center in Israel. All experiments were approved by the Institutional Animal Care and Use Committee, and performed according to OECD guidelines for chemical testing. One group of five mice (7683–87) received visabron *c* (4–4) by iv injection in a volume of 0.2 mL/mouse at a dose of 10 mg/mice (about 500 mg/kg), and another group of five mice (7688–92) received per-os visabron *c* (4–4) at a dose of 10 mg/mice, dissolved in a nanoemulsifying drug delivery system,^48^ in a bolus of 0.4 mL/mouse. After 48 h of exposure, the animals were sacrificed and the organs were harvested for the detection of possible toxicological lesions in the framework of a safety assessment. Then the organs were fixed in 4% formaldehyde, trimmed in a standard position per organ, and transferred to embedding cassettes. Paraffin blocks were sectioned at approximately 3–5 μm thickness. The sections were applied on a glass slide and stained with hematoxylin and eosin (H&E). Pictures were taken on a microscope (Olympus BX60) at magnifications of X4 and X10 using the microscope’s camera (Olympus DP73). An experienced pathologist (Dr. Loeb Emanuel) examined the slides. Microscopically findings were classified with standard pathological nomenclature and the severity of the findings was graded on a scale of minimal, mild, or severe. Grades of severity for microscopic findings were subjective; minimal was the least extent discernible and severe was the greatest extent possible.

#### DiscoverX’s SAFETYscan Methods

Screening of potentially significant off-target effects to binding and enzyme targets was performed via SafetyScreen44 offered by Eurofins Cerep-Panlabs. Visabron *c* (4–4) was tested at 1 and 0.1 mM. Compound binding was calculated as the percentage of inhibition of the binding of a radioactively labeled ligand specific for each target. The compound enzyme inhibitory effect was calculated as the percentage of inhibition of control enzyme activity. Results showing inhibition higher than 40% were considered to represent significant effects of the tested compound. Results showing inhibition lower than 25% as obtained with 0.1 mM Visabron *c* (4–4) were not considered significant and were mostly attributable to the variability of the signal around the control level. In each experiment, the respective reference compound was tested concurrently with visabron *c* (4–4), and the data were compared with historical values determined at Eurofins. The experiment was statistically accepted in accordance with the Eurofins validation standard operating procedure.

#### PhosphoSens CSox-Based Kinase Assays

Kinase activity was measured using the PhosphoSenstechnology (AssayQuant Technologies Inc., Marlborough, MA) as detailed in the Supporting Information.

#### Statistics

Each experiment was performed in triplicates. Unless otherwise stated, one-way ANOVA was performed using IBM SPSS software. In case of significance, Bonferroni posthoc analysis was performed. The results were considered significant when *p* < 0.05.

## Supporting Information Available

Purification and characterizations of visabres disintegrin, synthesis and characterizations of TMLD linear and cyclic peptides, visabron’pharmacokinetics, safety and supplemental methods (PDF)

## Notes

^#^ Part of a Ph.D. thesis to be submitted to The Hebrew University of Jerusalem

## Dedication

^†^ Dedicated to the memory of J.K., who enriched the life of all of us who were privileged to know and work with him.
