# Supplementary material for: Synthesis and Pharmacological Characterization of Visabron, a Backbone Cyclic Peptide Dual Antagonist of α4β1 (VLA-4)/α9β1 Integrin for Therapy of Multiple Sclerosis
Source: JACS Au. 2021 Nov 24;1(12):2361–76. doi: 10.1021/jacsau.1c00496 (PMC8717366; doi:10.1021/jacsau.1c00496)
Supplement: Supplementary file 1 — au1c00496_si_001.pdf [file au1c00496_si_001.pdf]

Electronic Supporting Information

This PDF file includes : supplementary figures, tables and methods; Figures S1 to S10; Tables S1 to S12.

Synthesis and pharmacological characterization of Visabron, a backbone cyclic peptide dual antagonist of α4β1 (VLA-4)/ α9β1 integrin for therapy of multiple sclerosis

Chaim Gilon[†], Michal Klazas[#*], Adi Lahiani[‡], Adi Schumacher-Klinger[#], Shira Merzbach[#], Johnny N. Naoum[†], Haim Ovadia[§], Limor Rubin[&], Susan Cornell-Kennon[+], Erik M. Schaefer[+], Jehoshua Katzhendler[‖**], Cezary Marcinkiewicz[¶], Amnon Hoffman[#] and Philip Lazarovici[‡*]

[†] Institute of Chemistry, The Hebrew University of Jerusalem, Jerusalem 91904, Israel

[#] Pharmacy, [‡] Pharmacology, and [‖] Medicinal Chemistry, Institute for Drug Research, School of Pharmacy, Faculty of Medicine, The Hebrew University of Jerusalem, Jerusalem 9112102, Israel.

[§] Neurology and [&]Allergy and Clinical Immunology Unit, Hadassah-Hebrew University Medical Center, Jerusalem, 9112001, Israel.

[+]AssayQuant Technologies Inc., 260 Cedar Hill Street, Marlboro, MA 01752, USA

[¶] Bioengineering, College of Engineering, Temple University, Philadelphia, PA, 19122, USA

**Correspondence to: philipl@ekmd.huji.ac.il*

Table of Contents **Page**

1. Purification and characterizations of Visabres disintegrin

1.1. Isolation of α4-disintegrin Visabres - Fig S1 3

1.2. Biological activities measured in FPLC fractions-results-Table S1 4

1.3. Assignment of FPLC and HPLC fractions to venom protein families by Mass Spectrometry-Table S2 and Table S3 5,6

1.4. Amino acid sequence determination of Visabres –Table S4 7

1.5. Antagonistic effect of Visabres on adhesion of α4β1 and α9β1 integrin- expressing cells, to immobilized VCAM-1-Fig. S2 8

1.6. Visabres' potency and selectivity for integrin antagonism in cell adhesion assays-Table S5 9

2. Synthesis and characterizations of TMLD linear and cyclic peptides

2.1. Synthesis of the Fmoc-[N-(Alloc)ω-aminoalkyl]glycine building units (Gly BU) used for the synthesis of the Visabron c(m-n) mini library -Fig. S3 10

2.2. Analytical HPLC analyzes of the synthesized cyclic peptides - Fig.S4 11

2.3. Mass spectrometry analysis of the synthesized cyclic peptides - Fig S5 12

2.4 Analytical HPLC and MS results of the synthesized backbone cyclic Visabron peptides –Table S7 13

2.5. Analytical HPLC and mass spectrometry analysis of Arg-Visabron c(4-4) Analog-Fig. S6 14
2.6. Sequence similarity between Visabron c(4-4) and CCK1-Fig.S10 15
2.7. cLogP of Visabron c(m-n) peptides-Table S8 15
2.8. TMLD- linear peptides inhibition of adhesion of integrin expressing cells to the respective immobilized ligands-Table S6 16

3. Visabron'Pharmacokinetics

3.1 Metabolic stability-Fig. S7 17
3.2 Plasma concentration-Fig. S8 17
3.3 PK/PD relationship-Fig.S9 18

4. Visabron'safety

4.1. Hematocrit cell counting and hemoglobin analysis-Table S9 19
4.2. Biochemical analysis of blood-Table S10 20
4.3. Histological pathological grades of organs slides-Table S11 21

5. Supplemental Methods

5.1. Phospholipase A2 22
5.2. Toxicity 22
5.3. Proteolytic activity 22
5.4. Hemorrhagic activity 22
5.5. Sequence characterization of ethylpyridylated Visabres subunits 22
5.6. Pharmacokinetics 23
5.6.1 Animals 23
5.6.2. Plasma Stability Studies 23
5.6.3. PK Study 23
5.6.4. Bioanalytical Method for the Detection of Vis-c(4-4) in Rat Plasma 24
5.7. Blood cell counting and biochemistry analyzes 24
5.8. Visabron c (4-4) safety using PhosphoSens- CSox based kinase assays
5.8.1. Kinase activity measurements 24
5.8.2. Recombinant kinases 25
5.8.3. Final reaction conditions 25
5.8.4. Data analysis 25

6. References 26

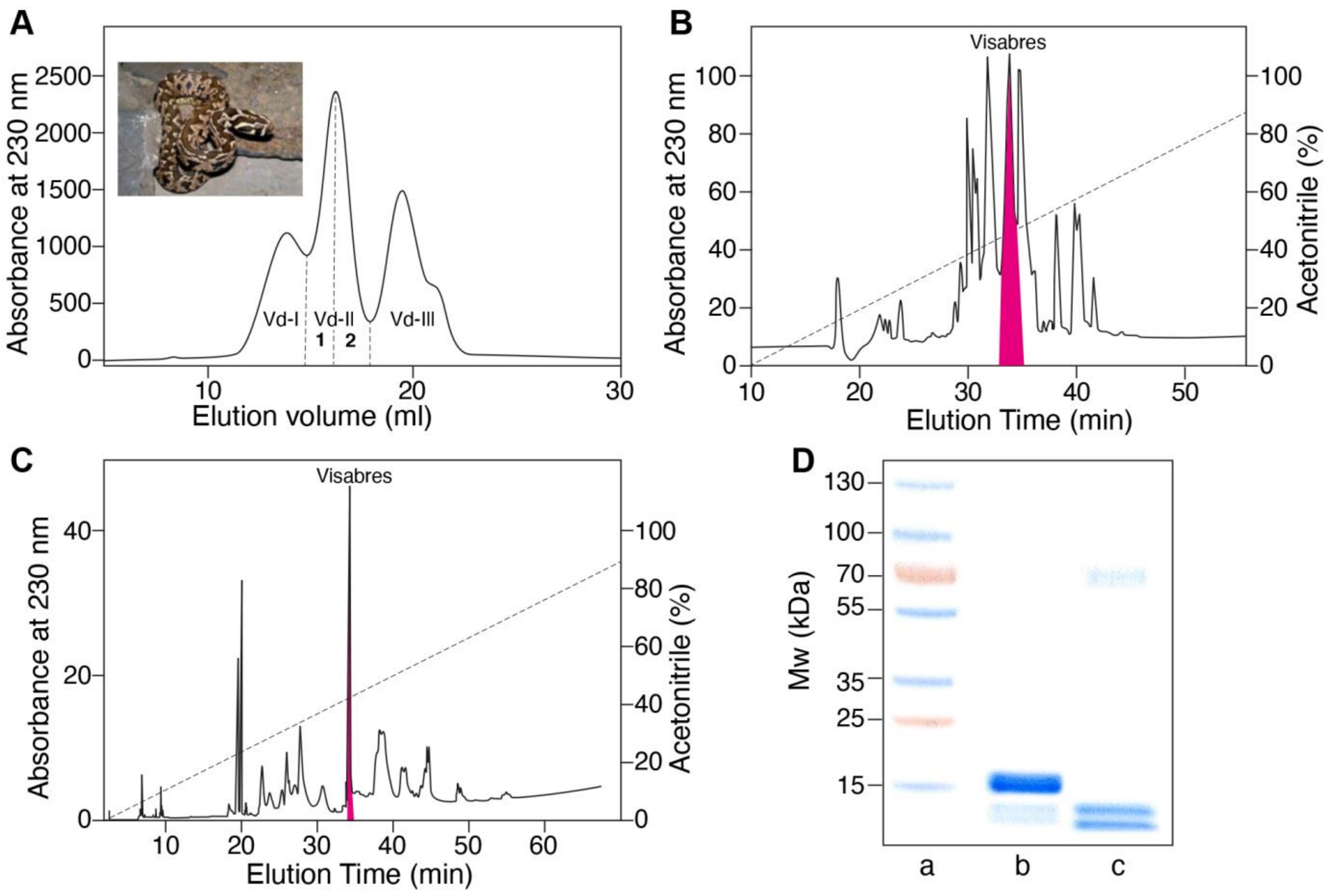

Figure S1- Isolation of α4-disintegrin Visabres from the venom of the Israeli snake *Vipera daboia (Vd).*

A. FPLC gel filtration of venom .Water extract of 500 mg/ ml venom was separated on a column of Superdex 200 (10/300 GL, GE Healthcare) using PBS (pH 7.4) at a flow rate of 0.5 ml per min. Separated fractions (with Vd numbers) were collected, dialyzed on bags with 1000 Daltons cutoff and lyophilized. **Insert** - photo of *Vipera daboia* snake.

B. Isolation of α4-disintegrin Visabres from the venom by reverse-phase HPLC. 100 mg of Vd-III fraction, in 200 µl of 0.1% TFA was injected into C18 column. The column was eluted with an acetonitrile-water gradient (0-85%, dashed lines) over 45 min. The fraction named Visabres, with anti- α4 integrin activity was collected and lyophilized.

C. To obtain higher purity, Visabres fraction was re-chromatographed over the same HPLC column, with a similar gradient (0-85 %, dashed lines) under similar conditions.

D. Visabres intact molecule (16 kDa) under non-reducing (b) and subunits A/B (8 kDa) under reducing conditions (c), after separation on 4-20% gradient gel using SDS -PAGE; Molecular weight markers (a).

Table S1- Biological activities measured in FPLC fractions of *Vipera daboia* venom

Phospholipase A_2	+	+++	+
α4-disintegrin antagonism	-	+/-	+++
Hemorrhagic activity	+++	++	-
Toxicity to mice	+/-	+++<br><br>LD_{50} = 0.8 µg / mice	+/-<br><br>LD_{50} = 15 µg / mice

Table S2 - Assignment of FPLC fractions to venom protein families by Mass Spectrometry [a]

Protein	Vd-I	Vd II-1	Vd II -2	Vd -III
Acidic PLA_2	+	+	+	+
Basic PLA_2	+	+		
Plasminogen activator		+	+	
Serine proteinase	+	+	+	
Zn^{2+}-metalloproteinase	+	+	+	
C-type lectin protein	+	+	+	
5'-nucleotidase	+	+		
L-amino acid oxidase	+	+		
Disintegrins			+	+
Snaclec (CRISP)			+	
NGF			+	
VEGF			+	
Serine protein inhibitor (Kunitz and other)			+	+

[a] Amino acid sequence similarity searches were performed by comparison to the available databanks using the BLAST program30 implemented in the WU-BLAST2 search engine at http://www.bork.embl-heidelberg.de.

Table S3- Assignment of the HPLC purified fraction- Visabres (Figure 1B) to venom proteins

**Accession-NCBI/TrEmbl**	**Description**	**MW [kDa]**
A0A1I9KNP8	Endogenous tripeptide metalloproteinase inhibitor (*Vipera ammodytes ammodytes*)	27.1
I2G9B4	Kunitz-type serine protease inhibitor PIVL (*Macrovipera lebetina transmediterranea*)	10.6
P00991	Kunitz-type serine protease inhibitor 1 (*Vipera ammodytes ammodytes*)	9.8
Q7LZ09	Lebetin-2-alpha (*Macrovipera lebetina*)	3.9
Q3BK14	Disintegrin lebestatin (*Macrovipera lebetina*)	11.5
P0C6E2	Disintegrin viperistatin (*Daboia palaestinae*)	4.5
A0A068EPZ2	Amine oxidase (*Gloydius intermedius)*	57.1
J3S9B2	Proactivator polypeptide-like (*Crotalus adamanteus)*	58.0
A0A0F7Z8W5	Tubulin alpha chain (*Crotalus adamanteus*)	49.9
B5U6Z2	Acidic phospholipase A_2 (*Macrovipera lebetina transmediterranea*)	15.4
Q6A3D6	Ammodytin I2(A) variant (*Vipera aspis aspis*)	15.2

Table S4- Amino acid sequence determination of Visabres disintegrin isolated from *Vipera daboia*[a,b]

A-subunit:

SGNPCCDPVYCKPRGEHCVSGPCCRNCKFLRAGTTCKRAVVDDMMDYCTGISSD CRRNPWKSE

B-subunit:

MNSANNPCCDPKTCKPRKGEHCVSGPCCRNCKFLLPGTICKR*TMLD*GLNDYCTG V(I/T)(T/S)(P/D)DCPRNPW(Y/K)SEEED

[a] Visabres molecule was reduced and cysteines were ethylpyridylated and the amino acid sequence of the subunits was established by a combination of mass spectrometry and N-terminal sequencing of its proteolytic fragments. Cysteine used for sequence alignment is underlined; TMLD a4-integrin binding motif is in italics; Amino acids with ambiguity are in parenthesis.

[b] B subunit sequence is tentative and needs to be completed and confirmed by DNA sequencing. MALDI-TOF mass spectroscopic analysis of intact Visabres yielded an apparent molecular weight of 16,850 Daltons, whereas subunit A and B indicated an apparent molecular weight of 8,160 and 8743 Daltons, each containing 10 ethylpyridylated cysteine residues, respectively.

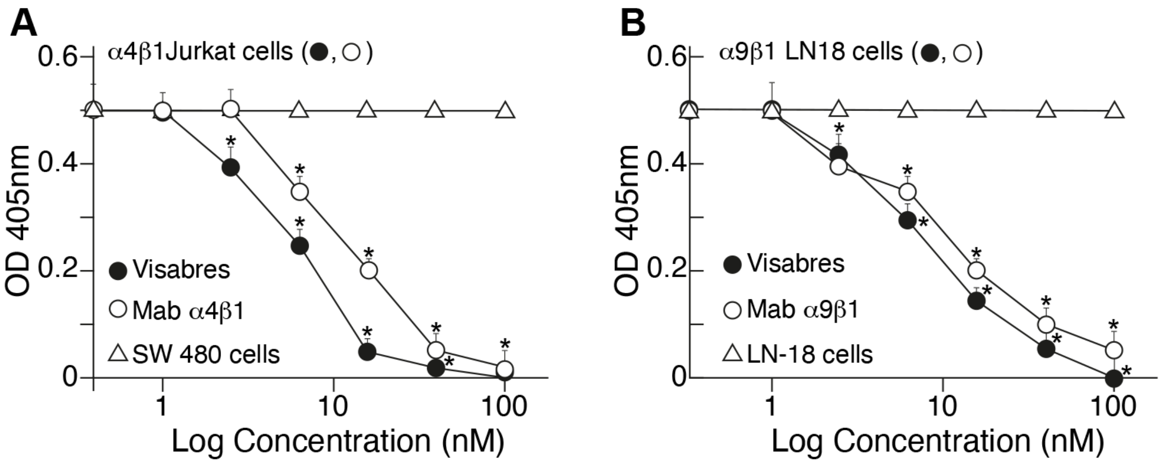

Figure S2- Antagonistic effect of Visabres on adhesion of α4β1 and α9β1 integrin-expressing cells to immobilized VCAM-1.

The inhibitory effects were determined in adhesion assay of CMFDA-labeled Jurkat cells (A) and α9β1-overexpressing, transfected-LN 18 cells (B) by comparison to control cultures SW480 and LN-18 lacking the integrins. Different concentrations of Visabres and monoclonal antibody (Mab) selective for either α4 (A) or α9 (B) were incubated in 100 µl of HBSS with calcium and magnesium for 30 min at 37°C with $1x10^5$ cells in 96 well plate immobilized with 5 µg/ml VCAM-1. After several washing with HBSS buffer the adhered cells were lysed by 1 % Triton X-100 and the plates were read with the spectrofluorometer.

Table S5- Visabres potency and selectivity for integrin antagonism compared to control VLO5 TMLD-disintegrin in cell adhesion assays

**Integrin**	**Ligand**	**Cell type in Adhesion Assay**[a]	**Visabres IC_{50} (nM)**	**VLO5**[c] **IC_{50} (nM)**
α1β1(ITGA1,CD49a)	Collagen-IV	α1-K562[b]	>5000	>5000
α2β1(ITGA2,CD49b)	Collagen-I	α1-K562[b]	>5000	>5000
α4β1(ITGA4,CD49d)	VCAM-1	Jurkat	5	5.5
α4β1(ITGA4,CD49d-ITGB1,CD29)	Recombinant human VCAM-1/CD106 Fc chimera protein	Ramos	6	2
α4β7(ITGA4,CD49d-ITGB7,CD49d)	Human recombinant MAdCAM-1/Fc chimera protein	RPMI 8866	170	135
α5β1(ITGA5,CD49e)	Fibronectin	K-562	>5000	>3500
α6β1(ITGA6,CD49f)	Laminin	α6-K562[b]	>10000	>10000
α9β1(ITGA9)	Tenascin C	α9- SW-480[b]	25	30
α1, α2, α5, α6	Tenascin C	SW-480	>5000	>5000

[a] K-562 = Human chronic myelogenous leukemia, SW 480 = Human colon adenocarcinoma, RPMI 8866 =Human lymphoblastoid from blood, Jurkat T-lymphocytes, Ramos (RA-1) B-lymphocytes, RPMI 8866 B-lymphocytes.

[b] integrin-overexpressor cells

[c] Similar value to those reported[1]

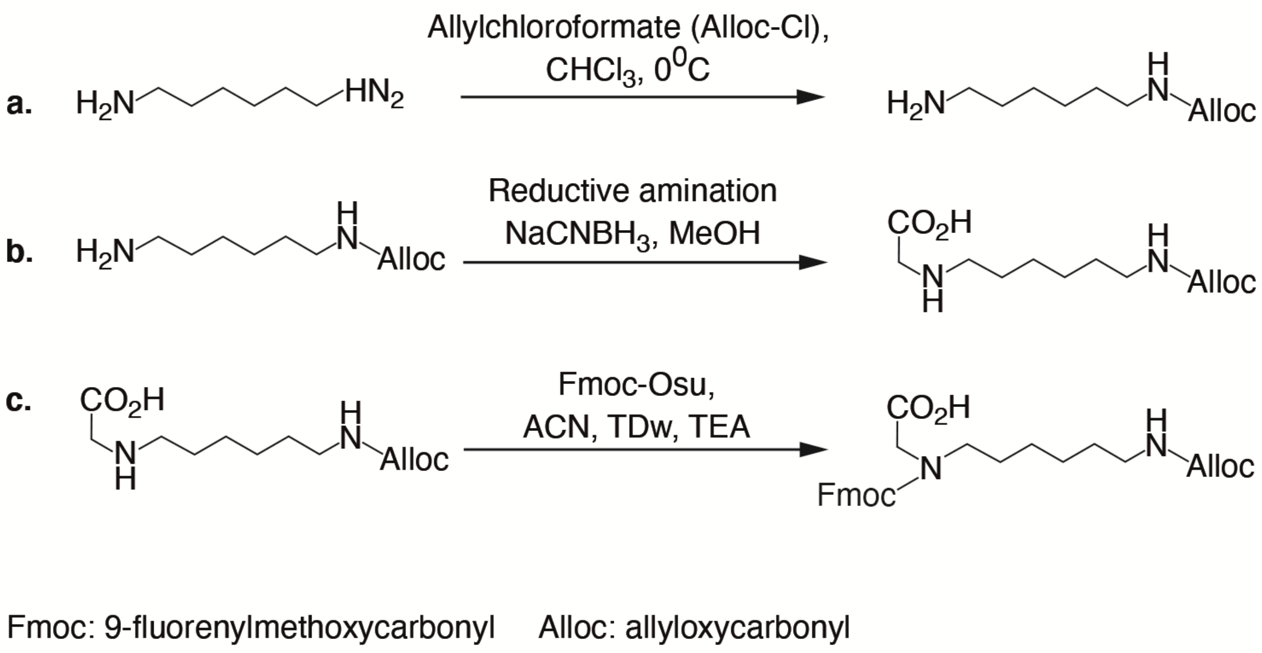

Figure S3- Synthesis of the Fmoc-[N-(Alloc)ω-aminoalkyl]glycine building units (Gly BU) used for the synthesis of the Visabron *c*(m-n) mini library.

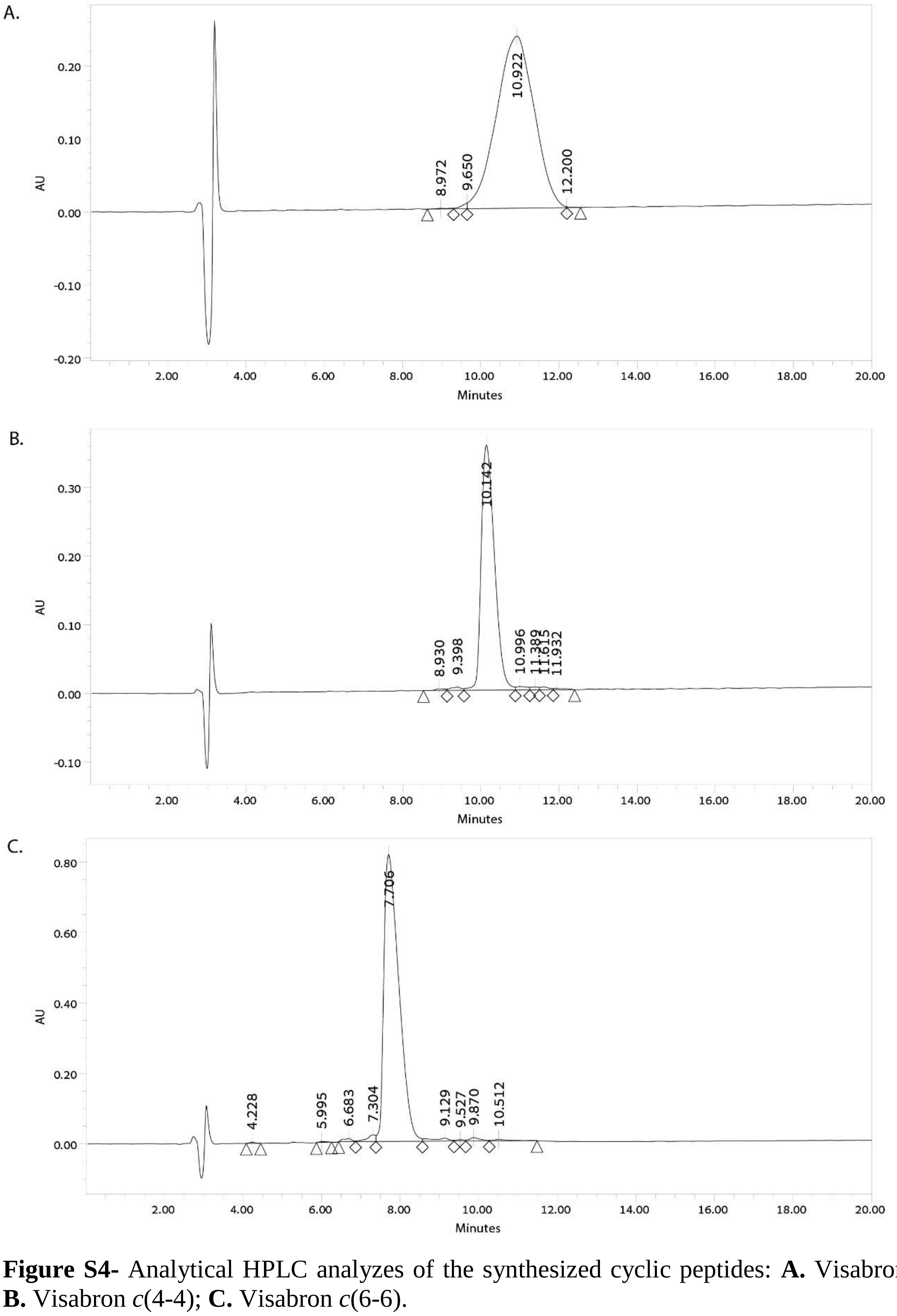

Figure S4- Analytical HPLC analyzes of the synthesized cyclic peptides: **A.** Visabron *c*(2-2); **B.** Visabron *c*(4-4); **C.** Visabron *c*(6-6).

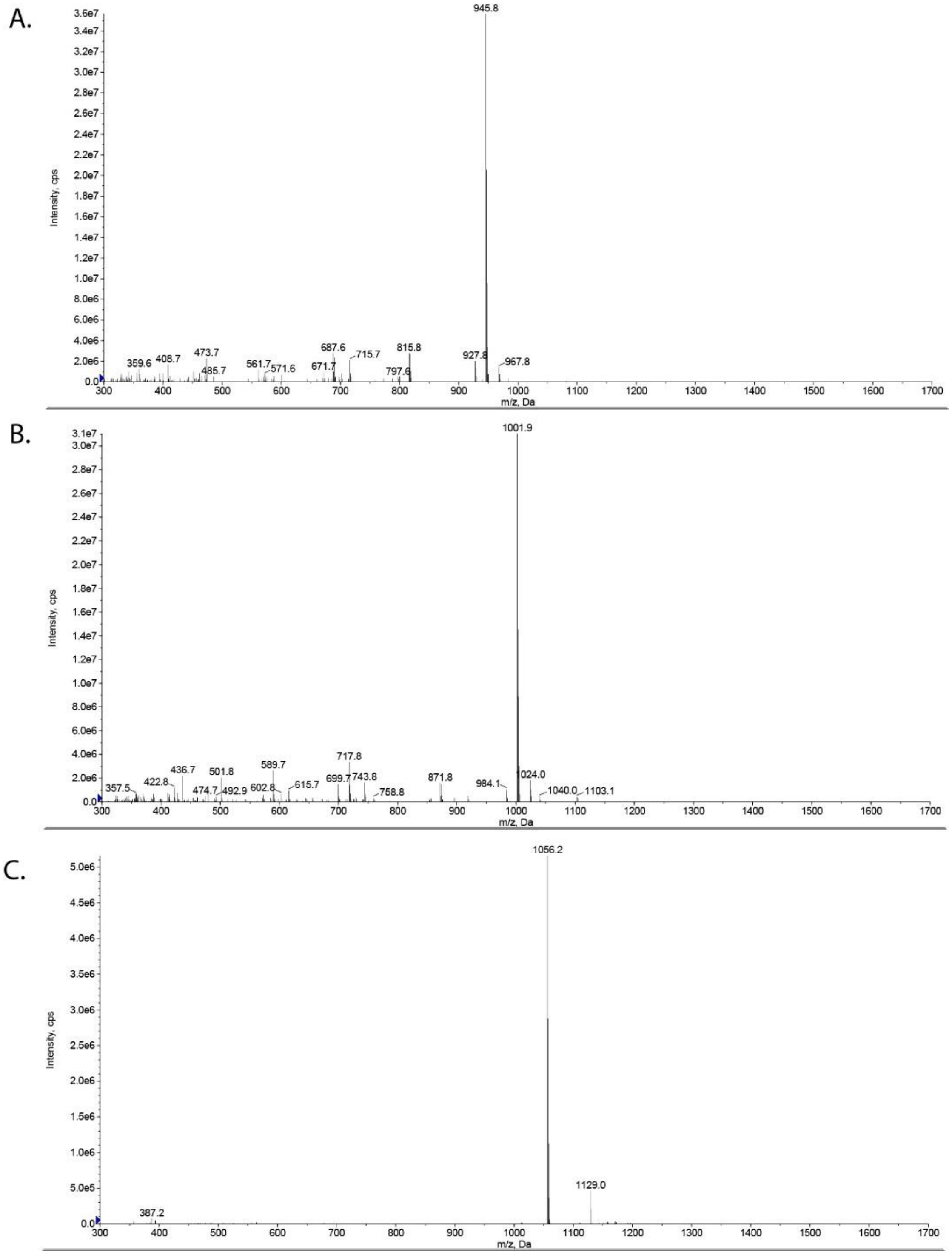

Figure S5- Mass spectrometry analysis of the synthesized cyclic peptides. **A.** Visabron <u>c</u>(2-2); **B.** Visabron *c*(4-4); **C.** Visabron *c*(6-6).

Table S7 - Analytical HPLC and MS results of the synthesized backbone cyclic Visabron peptides.

Peptide	Calculated M.W.	Observed M.W. (MS)	Purity (% Area from HPLC)
Vis *c*(2-2)	945.14	945.80	99.32
Vis *c*(4-4)	1001.24	1001.90	95.50
Vis *c*(6-6)	1057.35	1056.20	95.14

A

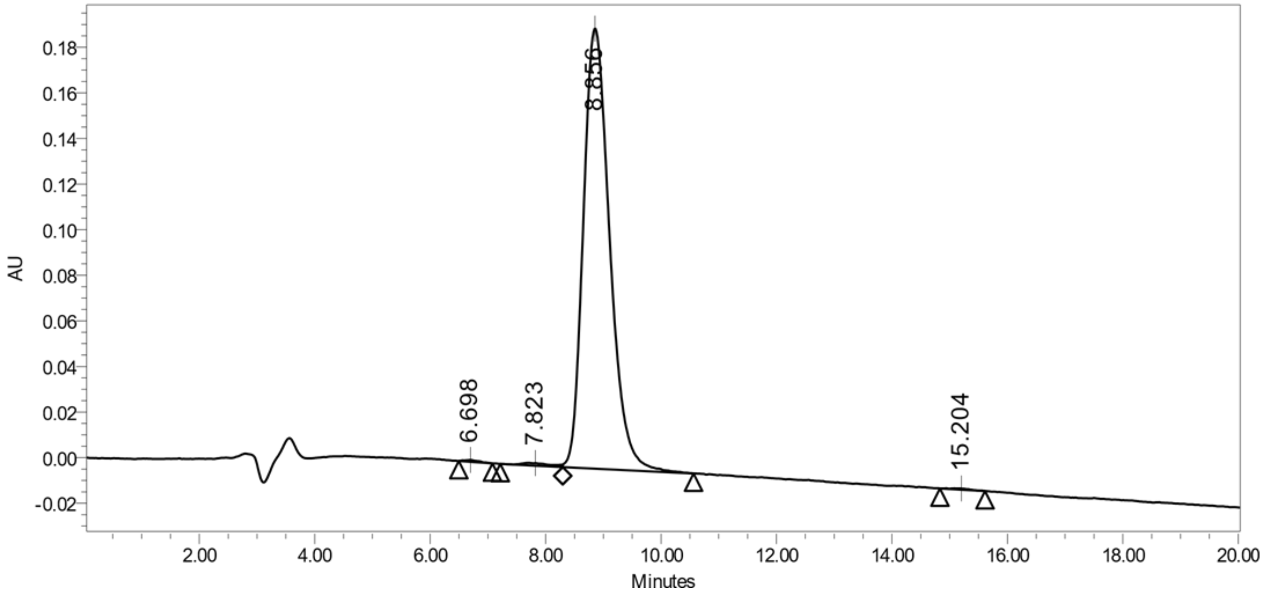

B

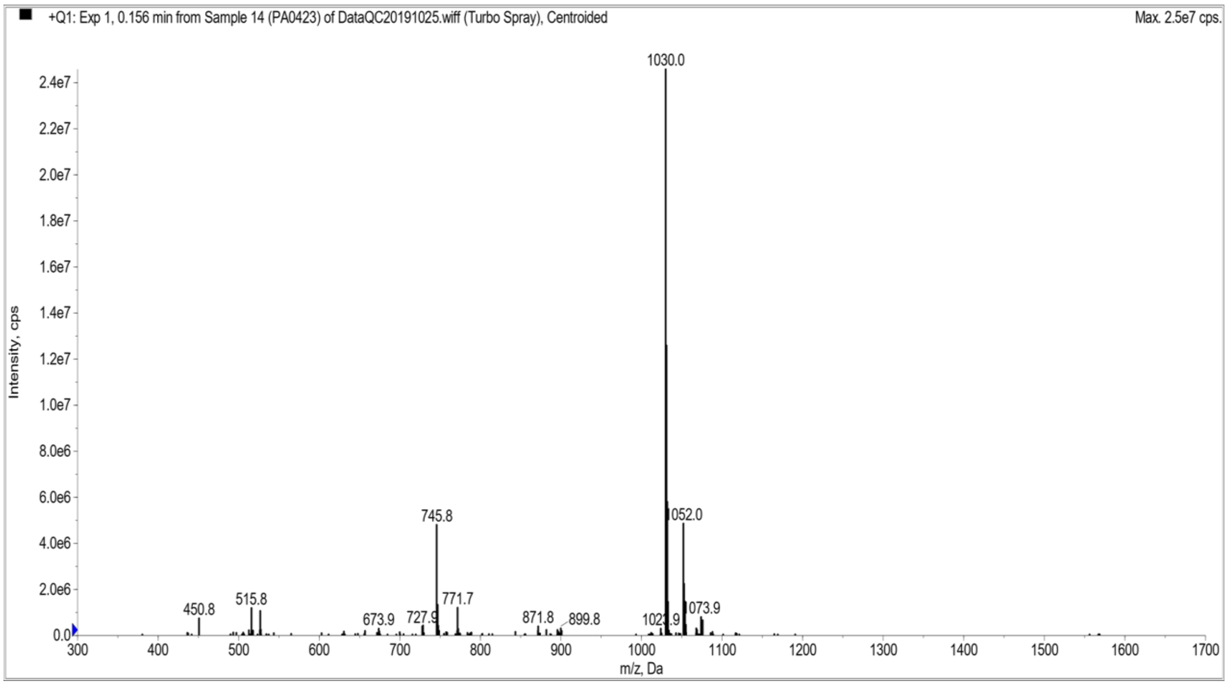

Figure S6- Analytical HPLC **(A)** and mass spectrometry analysis **(B)** of Arg-Visabron *c*(4-4) analog.

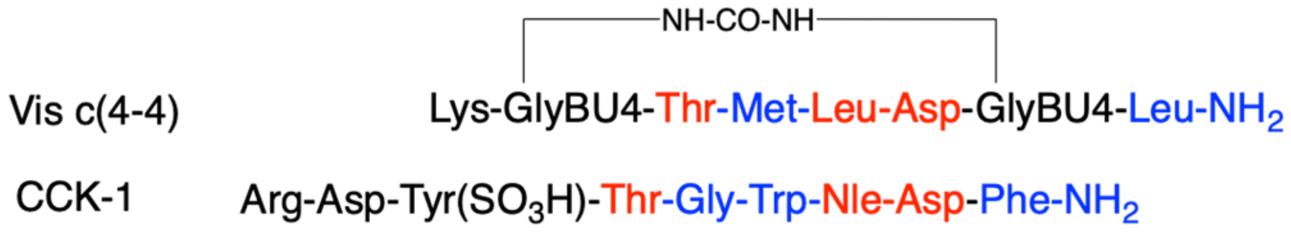

Figure S10. Sequence similarity between Visabron *c(*4-4) and CCK1.Amino acids in red are identical.Amino acids in blue are similar. This similarity can account for the binding of Visabron *c*(4-4) to the CCK receptors.

Table S8- cLogP of Visabron *c*(m-n) peptides

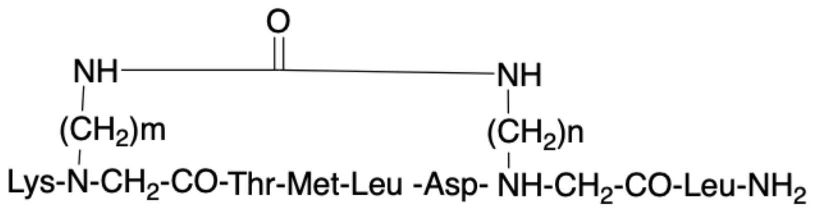

**Visabron *c*(m-n)**	**cLogP =**
Visabron *c*(2-2)	-2.2
Visabron *c*(4-4)	0.05
Visabron *c*(6-6)	2.3

Table S6. TMLD- linear peptides inhibition of adhesion of integrin expressing cells to the respective immobilized ligands [a]

	**Peptide Sequence**	**Size (number of amino acids)**	**α4β1[b]**	**α9β1[c]**	**α1β1[d]**	**α2β1[e]**	**α5β1[f]**
1.	C^{38}KRTMLDGLNDYC TGVTSDCPRNPWKSEEED^{68}	31	1550±95	1200±80	>10000	>10000	>10000
2.	CKRGLNDYC TGVTSDCPRNPWKSEEED	27	>10000	>10000	>10000	>10000	>10000
3.	CKRTMLDGLNDYC	13	1450±105	1250±95	>10000	>10000	>10000
4.	CKRAMLDGLNDYC	13	3910±80	2840±115	>10000	>10000	>10000
5.	KRTMLDGL	8	1310±95	1255±80	>10000	>10000	>10000
6.	*KG^{39}TMLDG^{44}L*	8	1550±125	1455±125	>10000	>10000	>10000

[a] The cells were incubated for 30 min at 37 °C with the cells (1 x 10^5) in the 96-well plate, previously covered with extracellular matrix ligand, in 100 µL of HBSS containing calcium and magnesium. After being washed with the same buffer, the adhered cells were lysed by Triton X-100, and the plate was read using Tecan spectrofluorimeter. The inhibitory dose 50% (IC_{50}) values representing mean ± standard deviations (n=6) were calculated from dose response curves. [b]Jurkat cells,VCAM-1; [c] α9-SW480 overexpressing cells, VCAM-1; [d] α1-K562 overexpressing cells, collagen IV ;[e] α2-K562 overexpressing cells, collagen I; [f] K562 wild type cells, fibronectin.

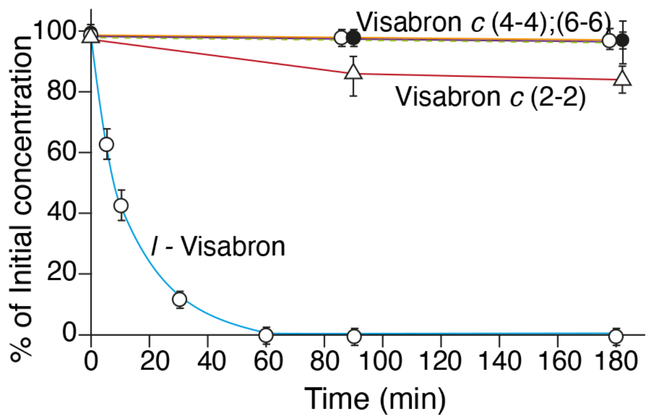

Figure S7- Metabolic stability of linear (blue) and cyclic Visabrons in rat plasma (n=3) (orange, green, red). The sequence of the linear Visabron is shown in Table S6 peptide #6. The amount of peptides is presented as a percentage of the sample at time 0.

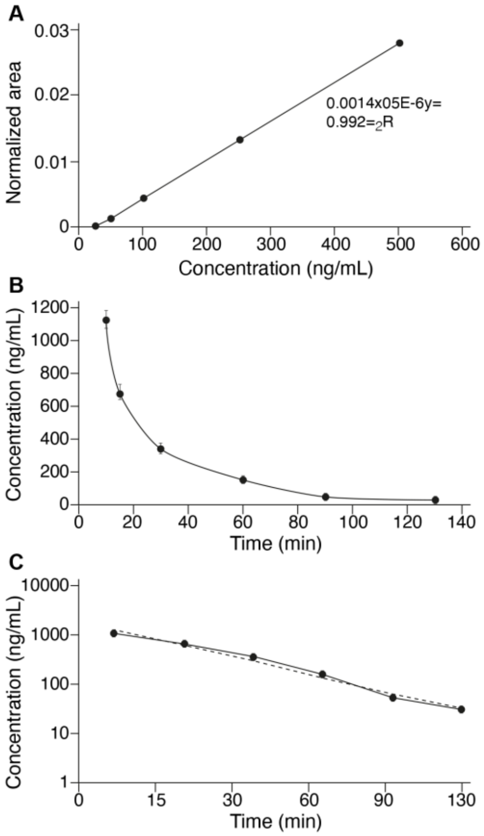

Figure S8- A. Calibration curve of Visabron *c*(4-4) (prepared by spiking the standard working stock solution with 15 µl of the different concentration, 20 µl metoprolol (10 µg/mL) and 135-µl blank rat plasma. **B.** Profile of plasma concentration of Visabron *c*(4-4) vs time in rats following i.v. bolus administration of 0.515 mg/kg; **C**. semi - logarithmic plot of plasma concentration of Visabron *c*(4-4) vs time (n = 4 , mean ± SD).

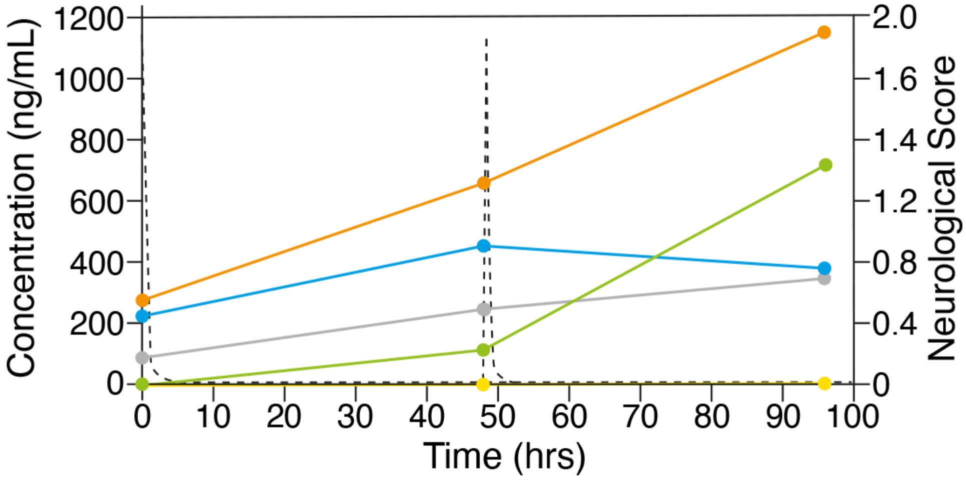

Figure S9- PK/PD graph representing the plasma concentration of Visabron *c*(4-4) and the EAE neurological scores vs time, observed from day 11 to 15 on the EAE experiments in mice. The plasma concentrations of Visabron *c*(4-4) were obtained following i.v. bolus administration of 0.515 mg/kg at time zero and again at 48 hours. The plasma of Visabron *c*(4-4) are presented by the dotted line seen following the two administration times in rats. The clinical scores were taken from the EAE experiments. Brown-PBS; Gray-Natalizumab; Yellow-Visabron *c*(4-4) 300 mg/kg; Blue- Visabron *c*(4-4) 90 mg/kg; Green- Visabron *c*(4-4) 25 mg/kg. This schematic presentation highlights the very different kinetics of Visabron *c*(4-4) in the blood that is eliminated rapidly while the pharmacology activity kinetics differ significantly (indirect PD).

Table S9 – Hematocrit cell counting and hemoglobin analysis of blood 24 hours after i.v. injection of 500 mg/kg of Visabron *c*(4-4) in C57BL/6 male mice.

**Parameter***	**Visabron *c*(4-4) (n=5)**	**Control (n=3)**
WBC (k/µL)	7.9±0.8	8.5±1.5
RBC (m/ µL)	9.0±0.9	9.5±1.2
HGB (g/dL)	14.8±0.5	15.5±0.7
MCH (pg)	14.8±0.6	14.1±0.9
MCHC (g/dL)	29±1.3	31±1.8
HCT (%)	45±2.5	46±2.7
MCV (fL)	48±2.5	51±2.8
MPV (fL)	5±0.9	5±1.4
RDW (%)	17.1±0.4	18.1±0.9

*WBC, whole blood cells; RBC, red blood cells; HGB, hemoglobin; MCH, mean corpuscular hemoglobin; MCHC, mean corpuscular hemoglobin concentration; HCT, hematocrit; MCV, mean corpuscular volume; MPV, mean platelet volume; RDW, red cell distribution width.

Table S10 – Biochemical analysis of blood 24 hours after i.v. injection of 500 mg/kg of Visabron *c*(4-4) in C57BL/6 male mice.

**Parameter***	**Visabron *c*(4-4) (n=5)**	**Control (n=3)**
BUN (mg/dL)	14±3.2	17.1±2.4
Calcium (mg/dL)	9.8±1.2	12±1.9
Chloride (mmol/L)	105±9.2	111±11
Cholesterol (mg/dL)	105±8.6	111±7.5
Albumin (gr/dL)	2.8±0.2	3.3±0.4
Creatinine (mg/dL)	0.3±0.07	0.4±0.09
Glucose (mg/dL)	279±31	260±42
AST (U/L)	92±11	98±8
ALP (U/L)	84±7	91±8
ALT (U/L)	41±4	48±6

BUN, blood urea nitrogen; AST, aspartate transaminase; ALP; alkaline phosphatase; ALT, alanine transaminase.

Table S11. Histological pathological grades of organs slides obtained from mice treated for 48 h with 500 mg/kg Visabron *c*(4-4)*

Animal Number**	Heart	Lung	Kidney	Small Intestine	Large Intestine***	Stomach	Spleen	Liver	Brain
**7683**	0	0	0	0	0	0	0	0	0
**7684**	0	0	0	0	3	0	0	0	0
**7685**	0	0	0	0	3	0	0	0	0
**7686**	0	0	0	0	0	0	0	0	0
**7687**	0	0	0	0	0	0	0	0	0
**7688**	0	0	0	0	0	0	0	0	0
**7689**	0	0	0	0	0	0	0	0	0
**7690**	0	0	0	0	2	0	0	0	0
**7691**	0	0	0	0	0	0	0	0	0
**7692**	0	0	0	0	0	0	0	0	0
**7693-7695**	0	0	0	0	0	0	0	0	0

*Grade 0: no signs of pathological changes; Grade 1: mild pathological changes; Grade 2: moderate pathological changes; Grade 3: severe pathological changes.
**Visabron *c*(4-4) treated 7683-7692; Control 7693-7695.
***In three mice, it was found in the last part of the colon, rectum and the adjacent anal unhaired skin a severe purulent, necrotizing inflammation. This finding was most probably not related to the tested compound and was considered as an accidental finding since was also found in a few control animals (data not shown), probably due to allergic food sensitivity.

Supplemental Methods

Phospholipase A2: Activity was determined by a commercial colorimetric assay purchased from Abcam Co. (Cambridge, UK) that provides an accurate and convenient method for measurement of secretory PLA2 (sPLA2) activity. This assay uses the 1, 2-dithio analog of diheptanoyl phosphatidylcholine which serves as a substrate for most PLA2s (e.g., bee and cobra venoms, pancreatic, etc.). Upon hydrolysis of the thio ester bond at the sn-2 position by PLA_2, free thiols were detected using DTNB (5,5'-dithio-bis-(2-nitrobenzoic acid) (www.abcam.com/ab133089).

Toxicity: Reed–Muench method[2] was used for determining LD_{50} (50% death after 48 h exposure to test compound), upon i.p. injection in BALB/c male mice, aged 40 days (20 gr).

Proteolytic activity: Venom fraction was analyzed for caseinase activity using casein-containing sodium dodecyl sulfate polyacrylamide gel (Bio-Rad, Hercules, CA) under non-reducing conditions. Briefly, 25 µl aliquots were run on a 7.5% casein-containing polyacrylamide gel at 200 V for 1 h; the gel was then removed from the plates and washed three times in 0.05 M Tris, 0.02% NaN_3, pH 7.5 and 2.5% Tween 80, for 30. The gel was successively washed in 0.05 M Tris, 0.02% NaN_3, 2.5% Tween 80, 1 mM $ZnCl_2$, and 5 mM $CaCl_2$ for 30 min at 22 °C. Finally, the gel was incubated in 0.05 M Tris pH 7.5, 0.02% NaN_3, 1 mM $ZnCl_2$, and 5 mM $CaCl_2$ overnight at 37 °C and then stained with 1.2 mM Coomassie brilliant blue (Serva, Heidelberg, Germany) for 1 h. Destaining was done with destaining solution containing 70% water, 20% methanol, and 10% glacial acetic acid. The bands were visible as light bands against a dark blue background. For the active fraction' protease specificity assay we used as substrate fibrinogen (Gibco, BRL) and fibronectin (Invitrogen Life Technologies, Carlsbad, CA). The assays was started by adding the venom fraction to the different substrates at the ratio of 40:1 (substrate: enzyme ratio; w/w) in 50 mM Tris, 10 mM NaCl, 2 mM $CaCl_2$, 1 mM $ZnCl_2$, pH 8.0. Substrates incubated without enzyme were used as controls. After 24 h of incubation, 20 µl of each preparation was mixed with electrophoresis sample buffer and the proteolytic reaction products were evaluated by SDS-PAGE. In order to determine the nature of the protease activity different protease inhibitors were used, namely: EDTA (10 mM), o-phenantroline (100 µM), leupeptin (10 µM). All the inhibitors were obtained from Sigma (Sigma Chemical Co., St Louis, MO).

Hemorrhagic activity: was assessed as described Theakston and Reid[3]. Venom and FPLC fractions at doses of 10 to 50 µg in 50 µL of saline solution were injected by intradermal route in BALB/c male mice. Hemorrhagic measurements were recorded after two hours. Diameters of hemorrhagic area were measured, and the minimum hemorrhagic dose (MHD) was defined as the venom dose that induced a lesion of 10 mm in diameter.

Sequence characterization of ethylpyridylated Visabres subunits: 1 mg/mL Visabres was solubilized in in 0.1 M Tris-HCl, pH 8.5, 4 mM EDTA, 6 M guanidine hydrochloride and reduced with 3.2 mM dithiothreitol for 3 h at room temperature in the dark. Reduced proteins were alkylated by addition of a 2-fold molar excess of 4-vinylpyridine over the reducing reagent. Ethylpyridylated (EP) A and B subunits were isolated by reversed-phase HPLC on a C-18 column. The isolated EP-subunits were submitted for N-terminal sequencing using an Applied Biosystems 477A and MALDI-TOF mass spectrometry using a PE-Biosystems Voyager-DE Pro instrument. The primary structures

of EP-polypeptides was deduced from the N-terminal sequence analysis of overlapping peptides, obtained by proteolytic digestions with trypsin (Sigma), endoproteinase Lys-C (Boehringer Mannheim), and endoproteinase Asp-N (Boehringer Mannheim) (2 mg/mL protein in 100 mM ammonium bicarbonate, pH 8.3, for 24 h at 37 °C using an enzyme: substrate ratio of 1:100 (w/w) and degradation with CNBr (10 mg/mL protein and 100 mg/mL CNBr in 70% (v/v) formic acid for 6 h at room temperature, under N_2 atmosphere and in the dark. Peptides were separated by reverse-phase HPLC, using a 4 × 250 mm C18 (5 µm particle size) Lichrospher RP100 (Merck) column eluting at 1 mL/min with a linear gradient of 0.1% TFA in water (solution A) and 0.1% TFA in acetonitrile (solution B). Visabres subunits were collected manually and dried in a SpeedVac (Savant). The purity and molecular mass of the reverse-phase-isolated proteins were checked by SDS/14% PAGE, N-terminal sequencing (using an Applied Biosystems Procise 492 sequencer), MALDI–TOF (matrix-assisted laser-desorption ionization–time-of-flight) MS using a Voyager-DE Pro instrument (Applied Biosystems), and 3,5-dimethoxy-4-hydroxycinnamic acid (sinapinic acid) (Sigma) saturated in 70% acetonitrile and 0.1% TFA as matrix, and electrospray ionization MS with a triple quadrupole-ion trap hybrid instrument (QTrap from Applied Biosystems) equipped with a Nano spray source (Protana). For peptide sequencing, the protein digest mixture was subjected to electrospray ionization MS/MS analysis using a QTrap mass spectrometer, equipped with a nanospray source. Doubly charged ions selected after Enhanced Resolution MS analysis were fragmented using the Enhanced Product Ion with Q0 trapping option at 250 atomic mass units/s (a.m.u./s) across the entire mass range. For MS/MS experiments, Q1 was operated at unit resolution, the Q1–Q2 collision energy was set to 35 eV, the Q3 entry barrier was 8 V, the LIT (linear ion trap) Q3 fill time was 250 ms, and the scan rate in Q3 was 1000 a.m.u./s. CID spectra were interpreted manually or using the online form of the MASCOT program (http://www.matrixscience.com). Quantitation of free cysteine residues and disulfide bonds was done as previously described[4].

Pharmacokinetics of Visabron c (4-4) following iv injection:

Animals-Male Wistar rats (275−300 g, purchased from Harlan, Israel), were used for the surgical procedure. An indwelling cannula was implanted into the right jugular vein, and exteriotrated at the dorsal neck to enable serial systemic blood sampling. After completion of the surgical procedure, the animals were transferred to cages to recover overnight (12−18 h). The animals had free access to water and food during the recovery period and throughout the entire experiment. All surgical and experimental procedures were approved by the Animal Experimental Ethics Committee of the Hebrew University, Hadassah Medical School, Jerusalem.

Plasma Stability Studies-Visabron *c*(4-4) (10 µg/mL) was mixed with fresh plasma from male Wistar rats (Harlan, Israel) and incubated at 37 °C for 180 min. Triplicate samples were taken at time 0 and after 5, 10, 15, 30, and 60 and 90 min . Samples (50 µL) were withdrawn and the reaction was terminated by adding 100 µL of ice cold acetonitrile (ACN). Each sample was spiked with metoprolol (IS, 5 µg/mL) and centrifuged (14635 g, 10 min). The supernatant was transferred to fresh glass tubes and evaporated to dryness. The glass tubes were reconstituted in 80 µL of mobile phase and centrifuged a second time (14635 g, 10 min).The reactions were analyzed using HPLC-MS.

PK Study-A single dose of Visabron *c*(4-4) in sterile water (300 µL of a 0.514 mg/mL solution, which was equivalent to a final dose of 0.514 mg/kg) was administered i.v. via the indwelling jugular cannula to each rat. Following dose administration, blood samples were taken at pre-dose, 10, 15, 30, 60, 90 and 130-min from the onset of the drug administration (time zero) via the right jugular vein. Approximately

350 µL of whole blood was removed, and placed in sodium-heparin containing tubes, and immediately placed in wet ice. Blood was centrifuged at 5322 g for 10 min and a 150 µL aliquot of plasma was transferred to fresh centrifuge tubes and stored at −20 °C pending analysis. PK analysis was performed using standard non-compartmental analysis models in Phoenix WinNonlin®.

***Bioanalytical Method for the Detection of Vis-c(4-4) in Rat Plasma*:** Plasma samples (150 µL) were spiked with metoprolol (20 µL, 10 µg/mL) as an internal standard. 200 µL of ACN was added to each sample and vortex-mixed for 1 min. The samples were then centrifuged (14635 g, 10 min), and the supernatant was transferred to fresh glass tubes and evaporated to dryness (Vacuum Evaporation System, Labconco, Kansas City, MO, USA). The glass tubes were reconstituted in 80 µL of mobile phase and centrifuged a second time (14635 g, 10 min). The amount of the compounds was determined using an HPLC−MS Waters 2695 Separation Module, equipped with a Micromass ZQ detector. The resulting solution was injected (10 µL) into the HPLC system. The system was conditioned as follows : Xterra MS C18, 3.5 µm, 100A, 100 x 2.1 mm column (Waters), an isocratic mobile phase of ACN : water supplemented with 0.1% formic acid (15:85, v/v) and a flow rate of 0.2 mL/ min at 35 °C was used. The limit of quantification the peptide was 25 ng/mL. Calibration curve was prepared by spiking the standard working stock solution with 15 µL of the different concentrations, 20 µL IS (10 µg/ mL) and 135 µl blank rat plasma in a centrifuge tube on the analysis day. The plasma calibration curve had a range of 25 ng/Ml to 2.5 µg/mL.

Blood cell counting and biochemistry analyzes: Male C57BL/6 mice were injected intravenously with 0.2 ml of peptide in a dose of 500 mg/kg. No immediate toxicity symptoms were measured and thereafter, acute tolerability was observed. Since the hematopoietic system is one of the most sensitive parameters to assess the toxicity of drugs in humans and animals, tail vein blood samples were taken from control (n=3) and 250 mg/kg Visabron *c* (4-4) injected mice (n=5), after 24 hours from injection and submitted for hematocrit cell counting and biochemical analysis. No significant differences in blood count and electrolytes of special importance are normal liver (ALP, alkaline phosphatase; ALT, alanine transaminase and AST, aspartate transaminase) functions and normal kidney (creatinine and blood urea nitrogen) functions, indicating sufficient organ integrity at the end of the experimental period. The data obtained was similar to hematological parameters reported for this mice strain[5], and clearly indicates lack of differences on kidney and liver parameters between the two mice groups.

Visabron *c* (4-4) safety by in vitro evaluation using PhosphoSens- CSox based kinase assays*:*

A powerful method was used to measure the activity of recombinant protein kinases using a homogeneous and continuous (kinetic) format, where the level of chelation-enhanced fluorescence (CHEF) that is directly proportional to the amount of phosphorylated, real-time sensors consisting of sulfonamido-oxine (Sox) chromophore, linked to a peptide or protein substrates of selective kinases[6]. This assay is ideal for elucidating drug mechanism of action and is increasingly being applied earlier in the drug development workflow, to address ''off target'' effects and/or the challenges and opportunities for next generation lymphocyte protein kinase inhibitors.

Kinase activity measurements: Kinase activity was measured continuously using the PhosphoSens® technology (AssayQuant Technologies Inc., Marlborough, MA) according to the manufacturers' recommendations. This one-step homogeneous assay format uses chelation-enhanced fluorescence via optimized substrate sensors containing the unnatural fluorogenic amino-acid Sox.

***Recombinant kinases*:** The source of individual protein kinases and the sensors used to monitor kinase activity are listed in the following Table S12.

Table S12

#	Kinase	Enzyme Type	Enzyme Source (Cat. #)	Concentration	Sensor (15 μM)
1	ABL1	Cytoplasmic Tyrosine Kinase	Carna (08-001)	2.5 nM	AQT0032
2	AXL	ReceptorTyrosine Kinase	Carna (08-107)	5 nM	AQT0101
3	BLK	Cytoplasmic Tyrosine Kinase	Carna (08-164)	10 nM	AQT0101
4	BTK	Cytoplasmic Tyrosine Kinase	Carna (08-180)	5 nM	AQT0101
5	CSK	Cytoplasmic Tyrosine Kinase	Carna (08-111)	20 nM	AQT0001
6	EGFR-wt	ReceptorTyrosine Kinase	BPS BS (40187)	8 nM	AQT0001
7	FGFR1	ReceptorTyrosine Kinase	Carna (08-133)	1 nM	AQT0001
8	HCK	Cytoplasmic Tyrosine Kinase	Carna (08-169)	2.5 nM	AQT0101
9	HER2 (725-YVMA-726)	ReceptorTyrosine Kinase	SignalChem (PV3805)	10 nM	AQT0001
10	IRAK1	Serine/Threonine Kinase	Carna (09-101)	1 nM	AQT0326
11	JAK3	Cytoplasmic Tyrosine Kinase	Carna (08-046)	2 nM	AQT0104
12	KIT	ReceptorTyrosine Kinase	Carna (08-156)	2.5 nM	AQT0104
13	LCK	Cytoplasmic Tyrosine Kinase	Carna (08-170)	2.5 nM	AQT0104
14	LYNa	Cytoplasmic Tyrosine Kinase	Carna (08-171)	2 nM	AQT0101
15	PYK2	Cytoplasmic Tyrosine Kinase	Carna (08-138)	5 nM	AQT0101
16	RAF (Y340D/Y341D)	Serine/Threonine Kinase	Thermo (PV3805)	5 nM	AQT0688
17	SRC	Cytoplasmic Tyrosine Kinase	Carna (08-173)	2 nM	AQT0104
18	SYK	Cytoplasmic Tyrosine Kinase	Carna (08-176)	0.5 nM	AQT0001
19	ZAP70	Cytoplasmic Tyrosine Kinase	Carna (08-177)	2 nM	AQT0001

***Final reaction conditions*:** Protein kinase activity was determined in 54 mM HEPES, pH 7.5, 1 mM ATP, 1.2 mM DTT, 0.012% Brij-35, 0.52 mM EGTA, 10 mM MgCl2, 1% glycerol, 0.2mg/ml BSA, and 15 μM Sox-based sensor. A panel of Sox-based sensors (AQTx) was used to measure the kinase activity of both tyrosine and serine/threonine kinases as summarized in **Table S12**. All components, except enzyme, were equilibrated to 30 °C prior to setting up reactions in 384-well, white flat round bottom polystyrene NBS microplates (Corning, Cat. #3824). Each reaction (25 μL final volume) consisted of 2.5 μL of Visabron *c*(4-4) (dissolved in ultrapure water), that was preincubated with 5.0 μL enzyme dilution buffer (EDB) (1x) or kinase (5x in EDB) for 10 minutes at room temperature, followed by the addition of 17.5 μL of reaction mixture containing ATP & C(Sx) Substrate (previously preincubated for 5 minutes at 30˚C). Plates were sealed using optically clear adhesive film (TopSealA-Plus plate seal, PerkinElmer, Cat. #6050185), applied with either a roller (VWR 60941-118) or a paddle (VWR 60941-128) to eliminate evaporation and resulting drift. Fluorescence intensity measurements were read kinetically every 2 minutes from the top for 120 or 240 minutes at 30 °C, with excitation and emission wavelengths of 360 nm and 485 nm, respectively, using a Synergy Neo2 multi-mode plate reader (Biotek Instruments, Winooski, VT).

***Data analysis*:** Fluorescence, determined with identical reactions but lacking purified enzyme or crude cell lysate was subtracted from the total fluorescence signal for each time point, with both determined in duplicate, to obtain corrected relative fluorescence units (RFU). Corrected RFU values then were plotted vs. time and the reaction velocity for the first ~40 min (initial reaction rates) were determined from the slope using GraphPad Prism (La Jolla, CA) with units of RFU/min.

References:

1. Bazan-Socha, S; Kisiel, D.G.; Young, B.; David, R.; Theakston, G.; Calvete, J.J.; Sheppard, D.; Marcinkiewicz, C. *Biochemistry*. **2004**, 43, 1639.

2. Reed, L.J.; Muench, H. *The American Journal of Hygiene*. **1934**, 27, 493.

3. Theakston, R.D.G.; Reid, H.A. *Bull World Health Organ.* **1983**, 61, 949.

4. Jua´rez, P.; Sanz, L., Calvete J.J. *Proteomics* **2004**, 4, 327.

5. Otto, G. P. ; Rathkolb, B.; Oestereicher, M.A.; Lengger, C.J.; Moerth,C.; Micklich, K.; Fuchs, H.; Gailus-Durner, V.; Wolf, E.; Hrabě de Angelis, M. *J. Am. Assoc. Lab. Anim. Sci*. **2016**, 55, 375.

6. Peterson, L. B.; Yaffe, M. B.; Imperiali, B. *Biochemistry*. **2014**, 53, 5771.
